# An epilepsy-causing mutation leads to co-translational misfolding of the Kv7.2 channel

**DOI:** 10.1186/s12915-021-01040-1

**Published:** 2021-05-21

**Authors:** Janire Urrutia, Alejandra Aguado, Carolina Gomis-Perez, Arantza Muguruza-Montero, Oscar R. Ballesteros, Jiaren Zhang, Eider Nuñez, Covadonga Malo, Hee Jung Chung, Aritz Leonardo, Aitor Bergara, Alvaro Villarroel

**Affiliations:** 1 Instituto Biofisika, CSIC-UPV/EHU, 48940 Leioa, Spain; 2 Present address: Department of Physiology, Faculty of Medicine and Nursery, UPV/EHU, 48940 Leioa, Spain; 3 Present address: Department of Cellular and Molecular Physiology, Yale University School of Medicine, New Haven, CT USA; 4 Centro de Física de Materiales CFM, CSIC-UPV/EHU, 20018 Donostia, Spain; 5 Department of Molecular and Integrative Physiology, University of Illinois at Urbana-Champaign, Urbana, IL 61801 USA; 6 Departamento de Física Aplicada II, Universidad del País Vasco, UPV/EHU, 48940 Leioa, Spain; 7 Donostia International Physics Center, 20018 Donostia, Spain; 8 Departmento de Materia Condensada, Universidad del País Vasco, UPV/EHU, 48940 Leioa, Spain

**Keywords:** KCNQ channels, Epilepsy, Co-translational folding, Calmodulin, IQ domain, Encephalopathy, Force profile analysis

## Abstract

**Background:**

The amino acid sequence of proteins generally carries all the necessary information for acquisition of native conformations, but the vectorial nature of translation can additionally determine the folding outcome. Such consideration is particularly relevant in human diseases associated to inherited mutations leading to structural instability, aggregation, and degradation. Mutations in the KCNQ2 gene associated with human epilepsy have been suggested to cause misfolding of the encoded Kv7.2 channel. Although the effect on folding of mutations in some domains has been studied, little is known of the way pathogenic variants located in the calcium responsive domain (CRD) affect folding. Here, we explore how a Kv7.2 mutation (W344R) located in helix A of the CRD and associated with hereditary epilepsy interferes with channel function.

**Results:**

We report that the epilepsy W344R mutation within the IQ motif of CRD decreases channel function, but contrary to other mutations at this site, it does not impair the interaction with Calmodulin (CaM) in vitro, as monitored by multiple in vitro binding assays. We find negligible impact of the mutation on the structure of the complex by molecular dynamic computations. In silico studies revealed two orientations of the side chain, which are differentially populated by WT and W344R variants. Binding to CaM is impaired when the mutated protein is produced in cellulo but not in vitro, suggesting that this mutation impedes proper folding during translation within the cell by forcing the nascent chain to follow a folding route that leads to a non-native configuration, and thereby generating non-functional ion channels that fail to traffic to proper neuronal compartments.

**Conclusions:**

Our data suggest that the key pathogenic mechanism of Kv7.2 W344R mutation involves the failure to adopt a configuration that can be recognized by CaM in vivo but not in vitro.

**Supplementary Information:**

The online version contains supplementary material available at 10.1186/s12915-021-01040-1.

## Background

Mutations at the *KCNQ2* gene underlie early-onset genetic epilepsy, with different clinical outcomes (including Benign Familial Neonatal Epilepsy, BFNE and Epileptic Encephalopathy type 7, EE7) [1–5]. This gene encodes for K_v_7.2 subunits of tetrameric voltage-dependent potassium (K^+^) selective channels, which, combined with K_v_7.3 subunits, underlie non-inactivating M-current. K_v_7.2/K_v_7.3 channels are enriched at the plasma membrane of the axon initial segment (AIS) and distal axons [6]. With their characteristic slow voltage-dependent kinetics of activation and deactivation, and voltage-dependent opening within the subthreshold range of action potential generation, they are critical for neuronal excitability [7]. A number of pathogenic variants cluster at key functional domains that are involved in voltage sensing, ion conduction, selectivity, gating, or stabilization of binding to the essential co-factor PIP_2_ [3, 8, 9].

Clusters of pathological variants are also found at the calcium responsive domain (CRD), located intracellularly following the pore gate [3, 10, 11]. The CRD is an autonomously folding hairpin domain formed by two antiparallel alpha helices [12, 13], named A and B [14], that run under the membrane adjacent to the voltage sensor [15]. These helices are recognized by calmodulin (CaM) [16–19], which confers calcium (Ca^2+^) sensitivity [13, 20–24].

Some mutations in the *KCNQ2* gene have been suggested to cause misfolding [25, 26], a mechanism that has been identified in many hereditary diseases [27, 28]. However, little is known on how pathogenic variants located at the CRD affect folding. In this work, we have explored how a K_v_7.2 mutation (W344R) located in helix A of the CRD, found in patients with hereditary epilepsy [29, 30], interferes with channel function. The data reveal that the key mechanism involves the failure to adopt a configuration that can be recognized by CaM in vivo but not in vitro.

## Results

### Functional characterization of the W344R mutation

**Fig. 1:**
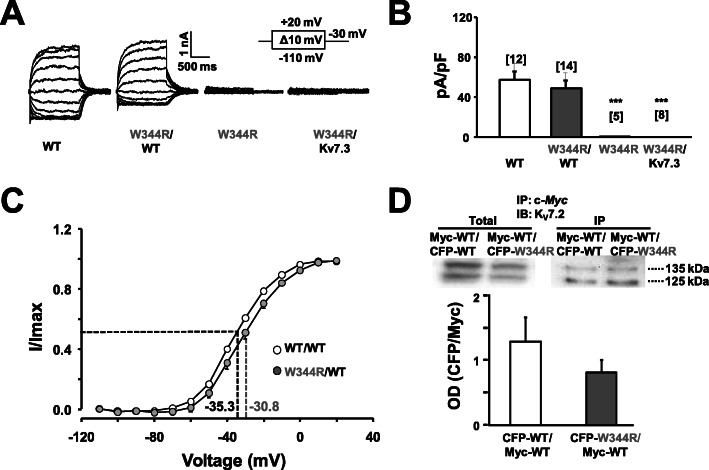
Functional consequences of the W344R mutation. **a** Representative current traces evoked in cells expressing the indicated subunits. Inset: voltage protocol. **b** Representation of the current density (pA/pF) from each group at + 20 mV from tail currents. K_v_7.2 wild type homomers (WT, white) and heteromers (WT/W344R, gray) had 57.0 ± 11.74 pA/pF and 49.0 ± 8.51 pA/pF, respectively. K_v_7.2 mutant (W344R) and K_v_7.2-W344R/K_v_7.3 heteromers produced negligible current. Number of cells is indicated in brackets. **c** Current-voltage relationship from tail currents measured at − 30 mV of K_v_7.2 WT homomers (*V*_1/2_ = − 35.3 ± 0.43 mV, *k* = 10.5; *n* = 12) and WT/W344R heteromers (*V*_1/2_ = − 30.8 ± 0.64 mV, *k* = 11.01; *n* = 14). ***p < 0.001. **d** Top: Representative immunoblot revealed with anti-K_v_7.2 antibody before (left) and after (right) immunoprecipitating with anti-Myc antibody from cells expressing the indicated Myc-tagged K_v_7.2 subunits (~ 125 kDa) and CFP-tagged K_v_7.2 (~ 135 kDa). Bottom: Relative densitometry of the two bands. IP, immunoprecipitation; IB, immunobloting. *n* = 3

The W344R mutation at the IQ site of helix A does not disturb CaM binding to the autonomously folding calcium responsive domain (CRD) of K_v_7.2 channels, yet it abolishes function [30]. This is in contrast to other helix A mutants, for which a clear correlation between CaM binding and function was observed [31] (Additional file 1: Figure S1). To understand this remarkable discrepancy, we re-evaluated its functional properties. In total agreement with previous work [29, 30], we found that homomeric W344R channels were not functional (Fig. 1a). The impact of this mutant in combination with its partner K_v_7.3 and wild type (WT) K_v_7.2 subunits (in a 1:2:1 ratio) to mimic genetic balance has been previously tested, revealing variable effects [29, 30]. To simplify the paradigm, we tested the impact of the K_v_7.2 mutant when combined with K_v_7.3 subunits in a 1:1 ratio (Fig. 1). K_v_7.3 alone yields negligible currents when expressed in HEK293T cells, whereas robust currents, that are two- or threefold larger than from homomeric K_v_7.2 currents, are recorded when combined with WT K_v_7.2 subunits in a 1:1 ratio (not shown).

No currents were observed in cells expressing K_v_7.3/K_v_7.2-W344R heteromers, but in contrast, robust currents were evoked for the K_v_7.2/K_v_7.2-W344R combination (Fig. 1a, b). There was a modification on the voltage-dependency, as revealed by a statistically significant ~ 5 mV rightward shift in the current-voltage relationship for the K_v_7.2/K_v_7.2-W344R arrangement (Fig. 1). Co-IP experiments were also consistent with the formation of heteromeric assemblies (Fig. 1d). HEK293T cells were co-transfected with Myc-tagged Kv7.2 WT subunits with either CFP-tagged WT or CFP-tagged mutant subunits in a 1:1 ratio. Protein lysates were immunoprecipitated with anti-c-Myc antibody and detected using anti-Kv7.2 antibody, resulting in the appearance of two bands due to the different MW imposed by the tags (Fig. 1d). No significant difference could be detected between the relative signal corresponding of Myc-WT (MW ~ 125 kDa) and CFP-tagged WT or W344R subunits (MW ~ 135 kDa).

**Fig. 2:**
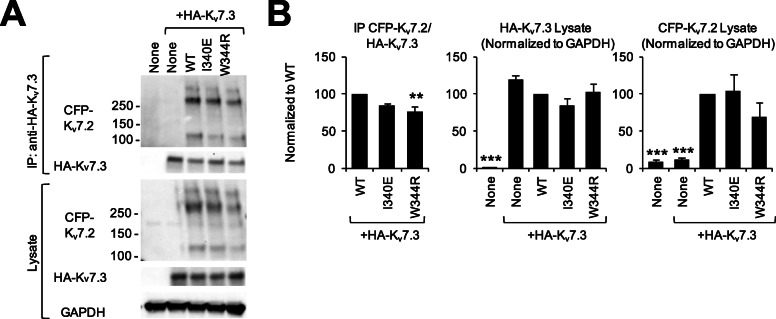
The W344R mutation modestly reduced HA-K_v_7.3 binding to CFP-K_v_7.2. Co-IP of HA-K_v_7.3 with K_v_7.2-WT, I340E, or W344R mutants with N-terminus CFP tag. **a** Representative immunoblot of Co-IP in HEK293T cells co-transfected with 1 μg of pcDNA3-HA-K_v_7.3 and 0.5 μg pcDNA3.1-CFP-K_v_7.2 WT or I340E, or 1 μg pcDNA3.1-CFP-K_v_7.2 W344R (see Additional file 1: Figure S2). The immunoblots show CFP-Kv7.2 monomers (~ 130 kD) and multimers, even though the immunoprecipitated fractions and lysates were treated with sodium dodecyl sulfate (SDS) sample buffer and the strong reducing agent TCEP. **b** Quantification of immunoblots (*n* = 5). GAPDH served as a loading control and all samples were normalized to WT with WT being 100%. Data represents the mean ± SEM (**p < 0.01, ***p < 0.005 vs CFP-K_v_7.2-WT)

In contrast, there was a slight, but statistically significant, reduction of the interaction of CFP-K_v_7.2 with HA-K_v_7.3 (Fig. 2). The interpretation of this result is complicated because protein levels for W344R were consistently lower than that of WT (49.8% ± 6.6% of WT, *p* < 0.005) or than that of the reference mutation I340E that precludes CaM binding. Aiming at getting similar protein levels, we halved the amount of CFP-K_v_7.2 WT or I340E plasmid transfected. The protein expression of W344R was still lower than that of WT or I340E (69.2% ± 19.2% of WT), although this difference was not statistically significant. After Co-IP, there was a statistically significant reduction of the signal for HA-K_v_7.3 when combined with K_v_7.2-W344R (76.43% ± 7.19% relative to WT, *p* = 0.01), but not when combined with K_v_7.2-I340E (84.34% ± 3.38%, p = 0.07) (Additional file 1: Figure S2A-B). Taken together, these results suggest that the W344R mutation modestly reduces the interaction of CFP-K_v_7.2 protein with HA-K_v_7.3. The basis of this reduction was not studied further, but may be related to the proposed reciprocal influence between CaM binding and tetramerization [32].

### The W344R mutation impairs trafficking

**Fig. 3:**
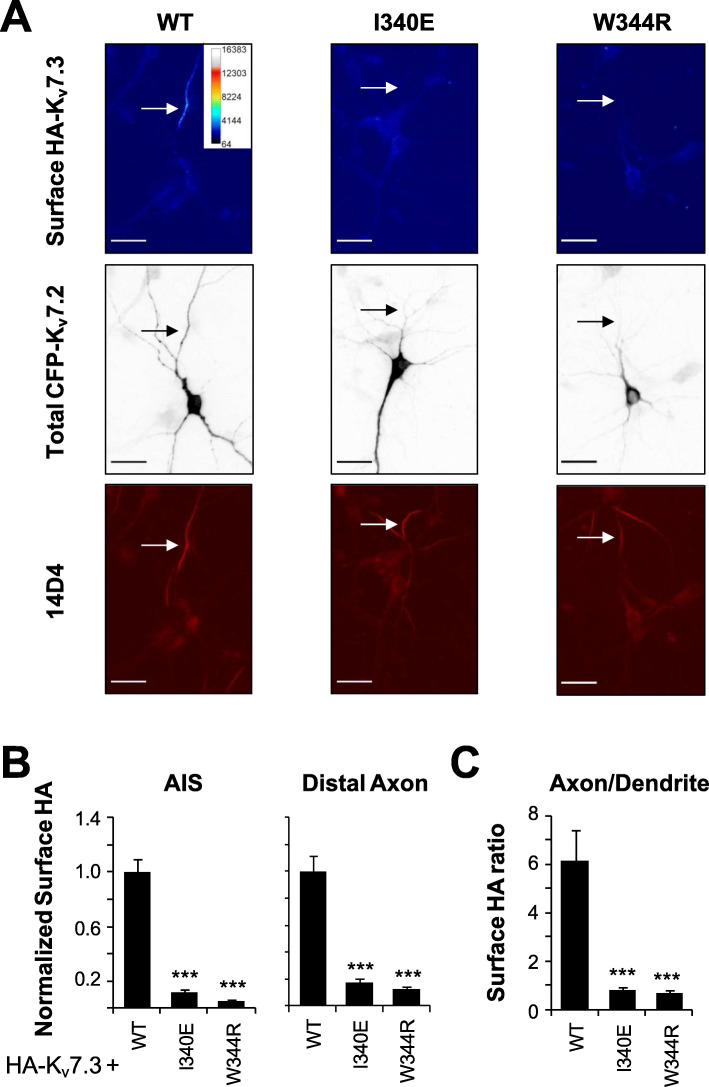
The W344R mutant severely reduced surface expression of heteromeric HA-K_v_7.3/K_v_7.2 in the axons of cultured hippocampal neurons. **a** Representative images of surface HA-K_v_7.3 as pseudo-color (upper panel), intracellular CFP-K_v_7.2 WT or mutants (middle), and the AIS marker 14D4 (bottom) from neurons transfected with HA-K_v_7.3 and CFP-K_v_7.2 WT, I340E or W344R. Pseudo-color represents different levels of surface HA-K_v_7.3 signal intensity as indicated by the calibration bar. Arrow indicates the locations of AIS. Scale bar, 25 μm (see Additional file 1: Figures S3-S4). **b** Background-subtracted fluorescent intensities of surface HA-K_v_7.3 from transfected neurons were normalized to those of HA-K_v_7.3/CFP-K_v_7.2-WT. **c** The axon/dendrite ratio was computed for surface HA-K_v_7.3/CFP-K_v_7.2 fluorescent intensities. Sample numbers for **b** and **c** are WT (*n* = 19), I340E (*n* = 14), and W344R (*n* = 13). ***p < 0.005

K_v_7.2 interaction with CaM is critical for the exit of K_v_7.2/K_v_7.3 channels from the endoplasmic reticulum (ER) and their expression at the axonal surface [6]. To assess the impact on trafficking to the axon initial segment (AIS) and other neuronal regions, surface immunostaining of K_v_7.3 subunits containing an extracellular HA epitope was evaluated [11, 33]. This K_v_7.3 reporter subunit was co-expressed in a 1:1 ratio with CFP-tagged K_v_7.2 WT or mutant W344R subunits in embryonic rat hippocampal neurons. As reference, the CFP-tagged K_v_7.2-I340E mutant was included because it precludes CaM binding [33, 34] and, as a consequence, it prevents the reporter K_v_7.3 subunit to traffic to the plasma membrane of different neuronal sub-compartments [33]. Plasma membrane expression of HA-K_v_7.3/K_v_7.2 WT heteromers were significantly higher at the AIS and distal axon compared to the soma and dendrite (Fig. 3, Additional file 1: Figures S3-S4). However, when K_v_7.2 carried the I340E or W344R mutations, the reporter HA-K_v_7.3 subunit failed to reach the AIS and distal axonal surface (Fig. 3). This lack of surface expression of HA-K_v_7.3/K_v_7.2-W344R channels (Fig. 3) and reduction in their complex formation (Fig. 2, Additional file 1: Figure S2) may underlie the absence of current in HEK293T cells (Fig. 1). The total signal for both mutant subunits was significantly reduced (Additional file 1: Figures S3 and S4). There were differences in surface expression at the soma, which may in part arise from homomeric K_v_7.3 channels that are expected to bind CaM. Except for the relative abundance at the soma, where the W344R but not I340E mutation decreased total CFP-K_v_7.2 expression by half (Additional file 1: Figures S3 and S4) similar to that in HEK293T cells (Additional file 1: Figure S2), the surface expression profile for W344R and I340E mutants were similar (Fig. 3), suggesting that neither bind CaM in a cellular in vivo context.

### The W344R mutation impairs calmodulin binding in cellulo

**Fig. 4:**
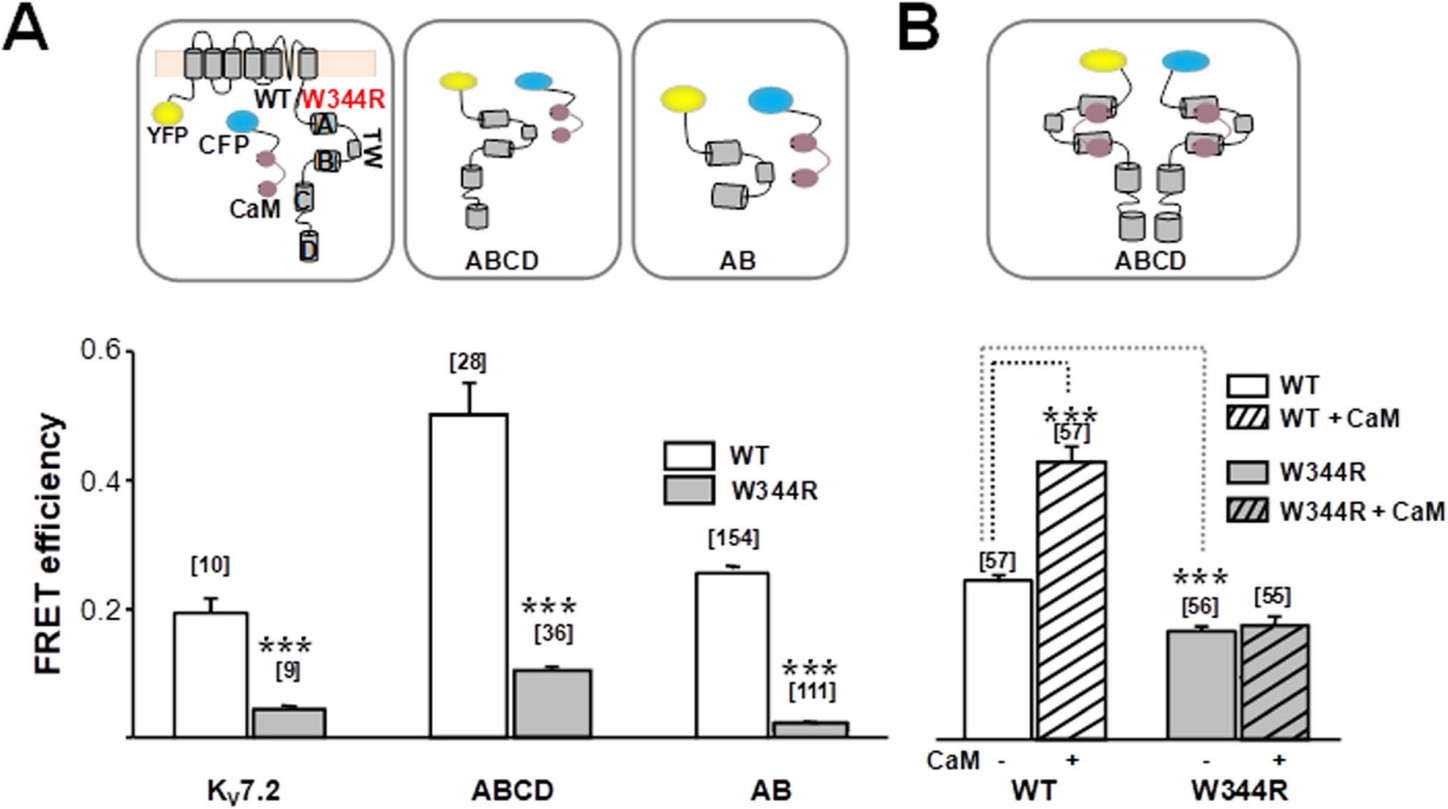
The W344R mutation disrupts K_v_7.2 interaction with CaM in living cells. **a** FRET efficiency from spectrally unmixed confocal images of HEK293T cells that express YFP-tagged channels or C-terminal domains (AB, ABCD) and CaM with an N-terminus mCFP-tag for the indicated configurations. There is an additional helix in the linker joining helices A and B referred to as TW. **b** FRET efficiency between ABCD domains before and after (dashed columns) CaM co-expression. Number of cells is indicated in brackets. ***p < 0.001

To assess CaM binding in cellulo, the transfer of energy between CaM and K_v_7.2 channels, tagged with CFP (donor) and YFP (acceptor) fluorophores, respectively, was monitored in living cells using Förster resonance energy transfer (FRET). This pair of fluorophores exhibits 50% energy transfer at ~ 50 Å and produces measurable transfer up to ~ 80 Å. Binding between a ligand and acceptor can be evaluated in cellulo using this approach, with comparable results to in vitro binding assays [35–37]. Cells expressing similar levels of donor and acceptor where included in the analysis (see “Materials and methods”). The ratio of the integral of CFP emission divided by the integral of YFP emission isolated after spectral unmixing of confocal images is proportional to FRET efficiency. Compared to WT channels, the FRET efficiency was significantly reduced for W344R subunits (0.18 ± 0.028 for WT vs 0.05 ± 0.005 for W344R, Fig. 4a).

Four alpha helices designated A through D can be recognized within the intracellular C-terminal domain of every K_v_7 channel [14, 38] (Additional file 1: Figure S5). CaM embraces the hairpin formed by helices A and B [12, 39, 40], which is joined by a flexible linker that contains a helix named TW [40]. The CRD is followed by two alpha helices, C and D, which run perpendicular to the membrane [15, 41]. Assembling as either heterotetramer or homotetramer depends on the identity of helix D [38].

To test if the W344R mutation decreases CaM binding to helices A and B of K_v_7.2 in cellulo, CFP-CaM was co-expressed with YFP-tagged C-terminal tail containing helices A through D that forms tetrameric complexes (YFP-ABCD), or with YFP-tagged helices A and B (YFP-AB) that forms monomeric complexes [35]. The transfer of energy was significantly larger when the acceptor was ABCD than when it was AB, and, importantly, the FRET efficiency was almost abolished for the W344R mutant proteins (Fig. 4a).

We have previously observed FRET in cells expressing helices ABCD (CFP-ABCD + YFP-ABCD) which is related to the ability of the D segment to form tetrameric coiled-coil assemblies [35]. The ABCD resembles a flower bouquet with the coiled-coil helix D corresponding to the pedestal [12]. The transfer of energy between these proteins increases with CaM overexpression (Fig. 4b), suggesting that CaM promotes a rearrangement of helix A within the ABCD region [35]. In contrast, the presence of the W344R mutation completely obliterated the FRET response to increased CaM expression, and this FRET response was lower under basal or elevated CaM conditions (Fig. 4b), suggesting that the C-terminal region adopted a more relaxed configuration due to the W344R mutation. These results reinforce the proposal that this mutation prevents CaM binding to the CRD of K_v_7.2 channels in living cells.

### The W344R mutation prevents proper folding during translation

**Fig. 5:**
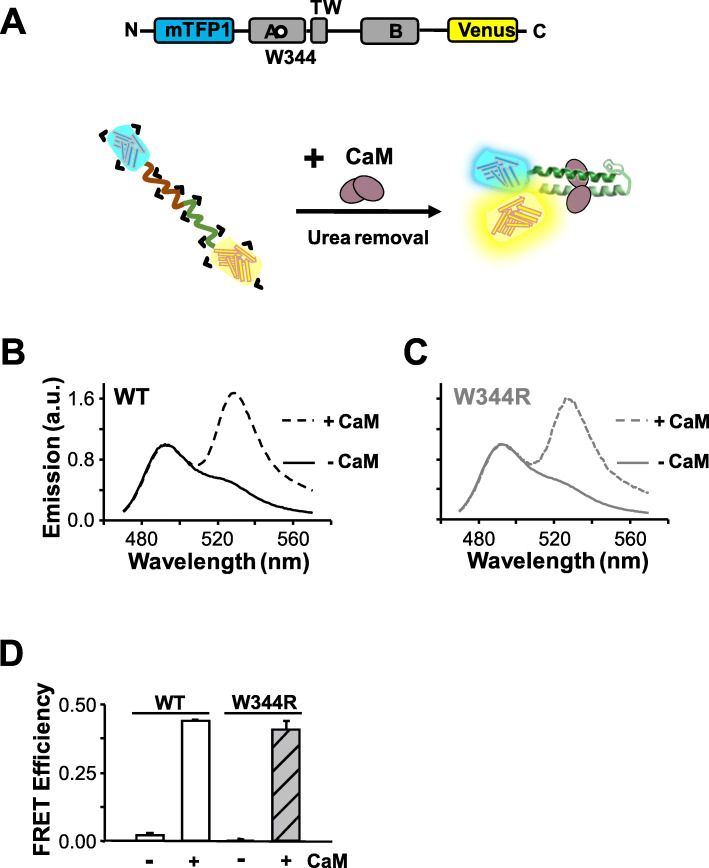
The W344R mutation is compatible with in vitro refolding of the CRD. **a** Top: Schematic representation of the “folding biosensor”. Helices A and B are flanked by blue mTFP1 and yellow mcpVenus fluorescent proteins. Bottom: Schematic interpretation of the experiment. Upon refolding in the presence of CaM, the fluorescent proteins are within FRET distance. Emission spectra of WT (**b**) and W344R (**c**) biosensors after denaturalization with 6 M urea and subsequent dialysis without (solid lines) and with (dashed lines) CaM. **d** FRET efficiency values of WT (white) and W344R (gray) proteins from data as in B and C. *n* = 3

We have previously shown that the W344R mutation does not disturb CaM binding to the GST-CRD fusion protein using in vitro binding assays, including dansylated-CaM fluorescence emission, far-Western, or surface plasmon resonance [30]. The discrepancy between in vitro and in cellulo CaM binding could be explained if the polypeptide follows different folding pathways. We hypothesized that the production and isolation of the GST-CRD fusion protein allowed folding in vitro of the CRD to a native configuration that could be recognized by CaM. To evaluate this, we used a “folding sensor” (Fig. 5a) which has been previously described [13]. This sensor contains helices A and B flanked by blue mTFP1 and yellow mcpVenus florescent reporters at the protein N- and C-terminal ends, respectively (Fig. 5a). Since helices A and B adopt an antiparallel fork configuration, co-expression of CaM brings both fluorophores close to each other, resulting in a FRET efficiency value of 0.40, computed from the ratio of peak emission at 528 and 492 nm. Addition of the strong chaotropic agent urea at 6 M reduced the FRET efficiency from 0.40 to 0. Since both mTFP1 and mcpVenus fluorogenic properties are not affected by this treatment, this FRET reduction indicates unfolding of the AB fork (Additional file 1: Figure S6).

**Fig. 6:**
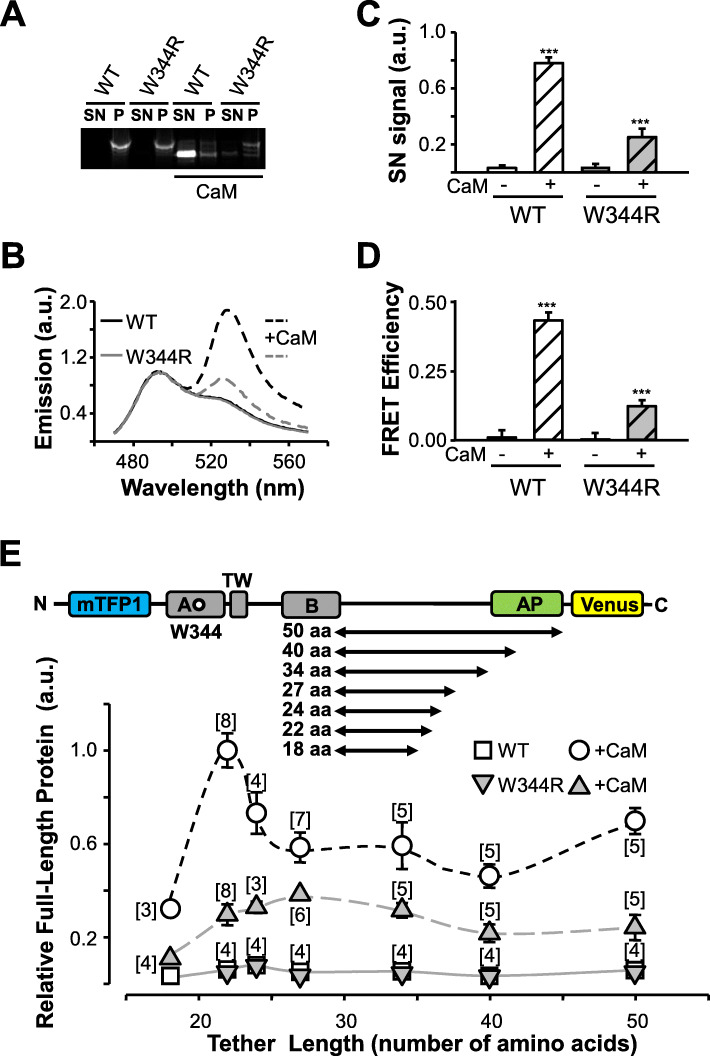
The W344R mutation disrupts CRD folding during translation. **a** Fluorescent image of an SDS-PAGE gel of unboiled bacterial extracts of WT or W344R biosensors expressed at 37 °C. Proteins were co-expressed (right columns) or not (left columns) with CaM. Soluble (supernatant; SN) and insoluble (pellet; P) protein fractions were separated, and loaded as indicated. **b** Fluorescence intensity of the supernatant band of SDS-PAGE gels (*n* = 3). **c** Emission spectra of the soluble fraction of WT (black lines) and W344R (grey lines) proteins expressed alone (solid lines) or co-expressed with CaM (dashed lines). **d** FRET efficiency values from spectra as in C (WT *n* = 16, WT + CaM *n* = 21, W344R *n* = 12, W344R + CaM *n* = 27). **e** Top: Schematic representation of the constructs used for in vivo co-translation folding monitoring. The CRD was cloned upstream of the SecM arresting peptide (AP) sequence with tethers of increasing length, ranging from 18 to 50 amino acids from the C-terminal conserved Pro of the SecM AP where translational stalling takes place. mTFP1 and mcpVenus were fused to the N- and C-terminus, respectively. Folding events of the protein domains inside or outside the ribosomal tunnel alleviate SecM stalling and leads to an increase in the ratio of peak emission mcpVenus/mTPF1. Bottom: Co-translational folding profiles of the indicated AB CRD constructs, expressed with and without CaM. Number of experiments is indicated in brackets

Folding in a cellular context in the absence of CaM was assessed in bacteria, since essential folding mechanisms are shared by prokaryotes and eukaryotes [28], and prokaryotes do not express CaM [42]. Most of the expressed folding sensor molecules were insoluble (Fig. 6a, left). The material from inclusion bodies was solubilized with 6 M urea, and thereafter the chaotropic agent was removed by dialysis in the presence or absence of CaM (Fig. 5b–d). The emission spectra from reconstituted WT and W344R folding sensors were indistinguishable, presenting FRET efficiency values congruent with proper folding when CaM was present (Fig. 5b–e). Thus, the W344R mutation is compatible with the adoption of the native AB fork configuration, consistent with the ability of CaM to bind in vitro to the AB protein [30].

The emission spectra of the small soluble fraction of either WT or W344R sensors translated in CaM-free bacteria did not display FRET (Fig. 6c), suggesting their unfolded nature. To test if the presence of CaM can induce their proper folding, excess purified CaM was added to the soluble fraction. However, no indication of CaM induced folding was observed as the FRET efficiency remained unaltered after up to 24 hours (Additional file 1: Figure S7), suggesting that the unfolded AB fork was stable and cannot be rescued by in vitro addition of CaM.

When CaM was co-expressed in bacteria and therefore present during translation, a larger fraction of the WT sensors became soluble (Fig. 6a, b) and presented a robust FRET (Fig. 6c). In contrast, for the W344R mutant, a large fraction ended up in inclusion bodies (Fig. 6a, b) although a small soluble fraction displayed significant FRET efficiency (Fig. 6d). This FRET efficiency represented 33% of that observed for the WT biosensor, suggesting that there was a small proportion of properly folded sensors carrying the W344R mutation.

To assess how the rate of translation affects the outcome, the same experiments were performed lowering the temperature to 18 °C during the induction of translation, but no significant difference was found compared to 37 °C (Additional file 1: Figure S8).

We exploited the ability of the SecM translational arresting peptide (AP) to act as a force sensor that detects folding of proteins in the ribosomal exit tunnel during translation [43–46]. The SecM AP interacts with the ribosome tunnel and detains protein synthesis, unless external force acting on the polypeptide chain “pulls-out” the AP, thereby relieving translational arrest [43]. Such pulling force can be induced by co-translational protein folding [43, 44], with equivalent results in vitro and in cellulo [45], and identifies the same co-translational folding transitions as do other methods, such as real-time FRET, photoinduced electron transfer, and NMR [47]. The K_v_7.2 CRD was cloned upstream of the SecM arresting peptide sequence with tethers of increasing length and flanked by blue mTFP1 and yellow mcpVenus fluorescent reporters in the N- and C-terminus, respectively (Fig. 6e). In this paradigm, if the protein is stalled, the result is an mTFP1-tagged truncated protein. In contrast, if CaM participates in folding of the CRD during translation and the full-length protein is translated, fluorescent signals from both mTFP1 and mcpVenus could be recorded (see Additional file 1: Figure S9). By measuring the stalling efficiency (as a fraction of full-length reporter protein) for a series of constructs of increasing tether length, it is possible to identify when the protein starts to fold during translation [46, 47].

Therefore, we used the ratio of emission at the peak wavelength for mcpVenus and for mTFP1 to assess the fraction of full-length reporter protein (Fig. 6e). The ratio was very low when WT or W344R AP reporters were expressed alone, but there was a robust signal for WT when expressed in the presence of CaM. This suggests that CaM exerts a fundamental role for CRD folding during translation. The index peaked for the WT construct with a tether of 22 residues, and then decreased as the length of the tether increased, reminiscent of profiles described for other proteins [45]. Although lower than WT, CaM also increased the translation of full-length W344R reporter, suggesting that a fraction of the W344R mutant reporter was folding during translation when CaM was present. In contrast to WT, a sharp peak could not be resolved for the W344R set. These results are concordant with FRET observed for the AB sensor (Fig. 6c, d).

### Two orientations for tryptophan and arginine at position 344

As a first approach to explore the stability of the W344R CaM/K_v_7.2-AB complex in silico*,* binding affinities were derived using the Rosetta Flex ddG software. Random changes in the backbone angles near the site of interest are introduced generating a trajectory of structures which are classified with respect to their resulting energy values. Afterwards, each of these random structures are ruled out or accepted, according to a Metropoli Montecarlo criteria, and resulting in small winning population of the energetically most stable configurations.

**Fig. 7:**
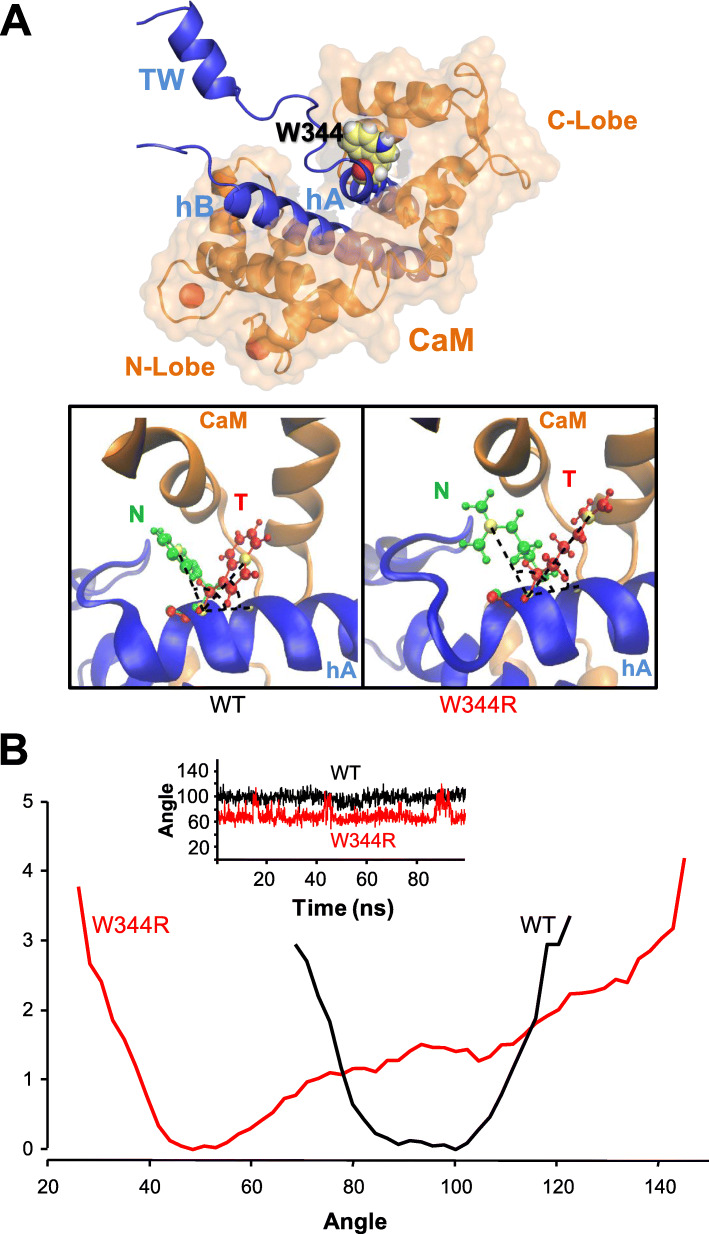
Two orientations for the lateral chain at position 344. **a** Top: Overall view of the CaM/AB complex with W344 in the N orientation (represented as filled stick, and colored using the Pymol atom coloring scheme). Bottom: Overlaid representation of both tilted (T, red) and native-like (N, green) structures of WT (left) and W344R mutant (right), where hA is blue whereas the CaM C-lobe is orange. Angles between hA and T or N structures are indicated. **b** Projection of the free energy in the angles between hA and tryptophan or arginine at position 344. Note that the angles of the N and T orientations depend on the presence or absence of CaM. Due to the mathematical computations involved, as WT is not able to explore angles in the ranges (20–65°) and (125–150°), the resulting free energy would be infinite, and it is not showed. The inset shows the time series of the angle through the simulation (Additional file 1: Figure S10)

We found that in most of the cases the side chain of tryptophan at position 344 is oriented towards CaM in the structures of the AB/CaM complex of different K_v_7 subunits [12, 13, 18, 39, 41]. Interestingly, detailed visualization of the final accepted trajectory snapshots of Rosetta moves revealed a small population (1.6% for WT and 3.4% for W344R) in which the side chain became tilted (T) towards the rest of the A helix, instead of targeting the CaM C-lobe, as it appears in the native (N) WT structure (Fig. 7a).

The computed value of the *ΔΔG* for W344R was 5.1 kcal/mol. This is a common value of a destabilizing mutation [3, 48, 49] (Additional file 1: Figure S10), suggesting that CaM binding to W344R should be disrupted. As binding does take place in vitro (Additional file 1: Figure S10), we wondered if Rosetta was failing to fully characterize a conformational change that would lower the *ΔΔG*.

To elaborate a hypothesis on the mechanism underlying the differential effect of the W344R mutation in vitro and in cellulo, we determined which orientation is the most stable in absence of CaM. For this purpose, a more accurate description of the forces involved is needed, so all-atom molecular dynamics (MD) simulations of the hA-TW-hB segment not engaged with CaM in a water cubic box for both WT and mutant were performed. After equilibration, the tryptophan residue at position 344 converges to the N position, whereas the arginine residue adopted the T orientation, and subsequent MD simulations were computed from these starting positions, respectively (Fig. 7b). In WT, tryptophan remains stable in the N configuration during 100 ns, and no transitions to the T state were observed. Furthermore, when W344 was forced to start from the T configuration, it transited to the N state in a few nanosecods and remained there, confirming that the N configuration is more stable (see Additional file 1: Figure S11). The energy needed to overcome the conformational change of the side chain from N to T is higher for the WT, which may underlie its inability to explore the T configuration. In W344R mutant, transitions between the two states were observed for the arginine residue, remaining more time in T (Fig. 7b), suggesting that the energy barrier between the two states in the absence of CaM is smaller. Since W344R remains for more time in T than in N, we conclude that the T configuration is the most stable when not engaged to CaM. To represent this information from an energetic perspective, the potential of mean force for the angles formed by the side chain and backbone were computed using the weighted histogram analysis method [50]. Figure 7b shows that the WT has a defined potential well around the N configuration. Conversely, W344R has its lowest energy configuration in T and the potential barrier between the two states is lower, so that it is more common to find the T configuration but the N is not energetically banned.

To identify which configuration of the mutated K_v_7.2 channel favors binding to CaM, the average of the non-bonding interaction energy between the residue W344R and CaM was computed using the CHARMM36 all-atom energy function for all the snapshots of Rosetta trajectories. In W344R mutant, the energy for T configuration was − 16 kcal/mol and for the N configuration was − 76 kcal/mol. In WT, the computed energies were − 10 and − 20 kcal/mol for the T and N configurations, respectively. These results suggest that the N configuration is more compatible with CaM binding than the T configuration and that arginine is more stable than tryptophan when helix A is in complex with CaM. The adoption of the T orientation favored by the W344R mutation may represent a kinetic trap in the path to a native fold [51]. This may explain the ability of both WT and W344R to bind CaM in vitro*,* although more data is required to fully test this hypothesis (see Additional file 1: Figure S10).

## Discussion

The analysis of mutations at helix A of the CRD of K_v_7.2 subunits has revealed an obvious relationship between CaM binding and function: subunits that do not bind CaM in vitro and in cellulo are retained at the ER, and channel function is concomitantly abolished [11, 31, 33, 34] (Additional file 1: Figure S1).

The W344R mutation found in patients with hereditary epilepsy [29, 30] is unique because it does not affect CaM binding to the CRD of K_v_7.2 in vitro [30] (Additional file 1: Figure S10), but it does abolish current and surface expression of K_v_7.2 channels and modestly perturbs its association with K_v_7.3 subunits (Figs. 1, 2, and 3). Two main hypotheses could explain this striking deviation from the common trend. One possibility is that this mutation may lock the gate of the channel in a closed state, for example, by impeding the expansion of the inner gate formed by the S6 bundle crossing that is directly attached to helix A. Alternatively, this mutation may prevent CaM binding within the cellular environment, but not in in vitro assays (Additional file 1: Figure S10).

Here, we show that the W344R mutation prevents FRET between CaM and K_v_7.2 subunits in cells. In addition, the tetrameric assembly of the four A-D helices in mutant K_v_7.2 [35] becomes insensitive to CaM abundance (Fig. 4). Therefore, the W344R mutation disrupts CaM binding to the isolated CDR or the full-length channel in living cells. Importantly, our FRET sensor that detects folding of the antiparallel AB fork, and the assessment of release from stalled ribosomes, revealed that the W344R mutation disrupts CRD folding during translation in the presence of CaM in cellulo (Figs. 5 and 6), indicating that the majority of the W344R mutant proteins fail to adopt a properly folded state during translation in living cells. This failure could result in their ER retention and rapid degradation, suggested by the decrease in their total expression in neuronal soma and HEK293T cells (Additional file 1: Figures S2-S4). Our results indicate that the presence of CaM during translation can be considered essential for proper folding of K_v_7 CRD, in agreement with previous proposals [52, 53]

What are the differential properties between tryptophan and arginine that lead to the failure of W344R to adopt a configuration that can be recognized by CaM in cellulo? We discovered two possible orientations (N and T) for the residue at position 344 (Fig. 7). When the CRD is not in complex with CaM, the adoption of the T orientation in WT CRD is very rare, concordant with atomic models [12, 13, 18, 39, 41], whereas the T configuration is stable and preferred over the N configuration for W344R CRD (Fig. 7). However, when helix A is in complex with CaM, the local interactions with the arginine residue in the W344R mutant are more favorable than the tryptophan residue in the WT (Fig. 7), consistent with in vitro binding data that demonstrates similar CaM interaction with WT and W344R CRD [30] (see Additional file 1: Figure S10).

We speculate that the vectorial nature of translation of the mutated K_v_7.2 channel, with the subsequent decrease on its available configurational space, could favor the T configuration of the W344R side chain during the transit of the nascent chain in the ribosomal tunnel. Consequently, after the nascent chain emerges from the ribosome in a eukaryotic cellular environment with abundance of CaM molecules, the W344R residue might remain in the metastable T configuration, which could prevent the proper binding between CaM and the mutated channel. In addition, this configuration may alter interactions with molecular chaperones or favor degradation. On the other hand, in vitro experiments might enlarge the accessible configurations for the W344R side chain, allowing it to find the most stable N configuration when interacting with CaM, favoring the mutated K_v_7.2 channel to properly bind CaM. The key difference is that in vivo the N-terminal portion can start folding before the C-terminal portion has been synthesized or is still within the ribosomal tunnel. In contrast, refolding of the full CRD in vitro can begin via interactions anywhere along the peptide chain [54]. In summary, the adoption of the T orientation favored by the W344R mutation may represent a kinetic trap in the path to a native fold [51], although more data is required to fully understand the process.

Genetic protein folding defects could be segregated into two categories: those due to mutations that are unsuited to adopt the native fold and those that can adopt the native structure with the use of chemical chaperons, low temperature, or ligands. For instance, the function of the N258S K_v_7.2 mutation, located in the extracellular turret just after S5, can be partially recovered with K_v_7.3 subunits, by culturing cells at lower temperatures or in the presence of the K_v_7-binding drug retigabine [26]. This second group could be divided further into those defects that lead to a weaker or unstable structure, and those that are compatible with a stable native configuration but fail to find the proper folding pathway. The W344R mutant fits readily in this latter category. We are not aware of any other pathological mutation with these properties and, therefore, this mutant may be the first representative within this group.

A remaining question is when and where does the W344R mutant deviate from the proper folding pathway. Some nascent chains can adopt an alpha helix configuration in the restricted space of the ribosomal tunnel and can fold into tertiary structures in its vestibule [28]. Analysis of the amino acid sequence of helix A using the Agadir server predicts that both WT and W344R will fail to form a stable alpha helix in solution, with scores of 0.4 and 0.7 (in a 0 to 100 scale), respectively. However, even unstable peptides may form helical structures in some regions of the ribosomal tunnel [55–57]. The vectorial nature of protein synthesis, the spatial constrains and physicochemical properties of the ribosomal tunnel, can guide the folding trajectory of the nascent peptide [28]. Our data and other observations are consistent with the idea that the emerging helix A segment, located at the N-terminus of the CRD, starts to fold before the C-terminal part of the protein is synthesized [58], and will initiate interactions with the CaM C-lobe in the vestibule or outside the ribosome during translation. It is not known if the emerging segment is already folded or if CaM induces the adoption of an alpha helix. Our data is compatible with the idea that CaM fails to promote alpha helix formation to the mutant W344R nascent chain, whereas in vitro binding is best described by selection of the properly folded molecules [59–61].

## Conclusions

In summary, we show here that the autonomously folding calcium responsive domain (CRD) carrying the W344R mutation is not recognized by CaM in a cellular in vivo context, but it does so after refolding in vitro. This mutation impedes proper folding during translation within the cell by forcing the nascent chain to follow a folding route that leads to non-functional ion channels. Thus, although it carries all the information for the native 3D configuration, it fails to reach it in vivo. Our MD simulations suggest a reasonable hypothesis for the underlying mechanism. Thus, this study provides a mechanistic insight into co-translational folding defects, which may represent a widespread mechanism that contributes to pathophysiology.

## Material and methods

### Cell culture and transfection

Human Embryonic Kidney cells (HEK293T) were cultured in DMEM (Dulbecco’s modified Eagle’s medium), supplemented with 10% of fetal bovine serum, 1% no-essential amino acids, and 1% of gentamicin. Cell cultures were maintained in 5% CO_2_ at 37 °C. Cells were transiently transfected with desired cDNAs using polyethylenimine (1 μg/μl; Polysciences) for electrophysiological studies, FRET, and co-immunoprecipitation experiments.

### Electrophysiology

The human isoform 3 K_V_7.2 (Y15065) and K_V_7.3 (NM004519) cDNA were tagged at the N-terminus with mCFP or mYFP fluorescent proteins respectively, and cloned into pcDNA3.1. The total amount of transfected cDNA was the same for all conditions except where noted. N-terminal tags have no impact on the electrophysiological properties of the expressed channels [5, 25]. Macroscopic currents were recorded at room temperature (22 °C) in the whole-cell configurations of the patch clamp technique using HEKA patch clamp EPC8. Borosilicate capillary glass (Sutter instrument) was pulled obtaining a tip resistance of 1–3 MΩ after filled with the internal solution. This solution contains (in mM) 125 KCl, 10 Hepes (K), 5 MgCl_2_, 5 EGTA, 5 Na_2_ATP adjusted to pH 7.2 with KOH, and the osmolarity adjusted to ~ 300 mOsm with mannitol.

Following patch rupture, whole-cell membrane capacitances were measured from integration of the capacitive transients elicited by voltage steps from − 50 to − 60 mV, which did not activate any time dependent membrane current. Series resistances were compensated 80% in order to minimize voltage errors and were checked regularly throughout the experiment to ensure that there were no variations with time. The voltage-clamp experimental protocols were controlled with the “Clampex” program of the “pClamp” software (Molecular Devices). HEK293T cells were perfused with the external solution containing (in mM) 140 NaCl, 4 KCl, 2 MgCl_2_ (6 H_2_O), 10 Hepes-Na, 2 CaCl_2_ and 5 glucose, pH 7.4 with NaOH, and the osmolarity adjusted to ~ 320 mOsm with mannitol.

The amplitude of the K_v_7 current was defined as the peak difference in current relaxation measured at − 30 mV after 500–1500 ms pulses to − 110 mV (all channels closed) and to + 20 mV (all channels opened).

### Co-immunoprecipitation (Co-IP)

A confluent T-75 flask of HEK293T cells were co-transfected with 5 μg of KCNQ2-WT cDNA in pcDNA3.1, N-terminally tagged with either mCFP or Myc, and 5 μg of CFP-KCNQ2-W344R cDNA for Co-IP experiments to analyze protein-protein interaction. Twenty-four hours after transfection, HEK293T cells were solubilized for 30 min at 4 °C in RIPA buffer, containing (mM) 20 Tris-HCl (pH 7.5),150 NaCl, 5 EDTA, 1% NP40 and protease inhibitors (1X Complete; Roche Applied Science). The lysate was centrifuged at 800×*g* for 15 min and the insoluble material was removed after centrifugation at 13,000 g for 15 min, after which the lysate was precleared for 1 h at 4 °C with 40 μl of equilibrated protein G-Sepharose beads (GE Healthcare). The day before, anti-*c-*Myc (Sigma-Aldrich) antibody was immobilized overnight at 4 °C with 40 μl of equilibrated protein G-Sepharose beads and washed six times with RIPA buffer. Precleared lysates were incubated overnight at 4 °C with protein G-antibody mix. After 6–8 washes with RIPA buffer, the immunoprecipitated proteins were released by heating at 90 °C 5 min in SDS sample buffer and were probed with anti K_v_7.2 antibody.

At 20–24 h post splitting, HEK293T cells were transfected with plasmids containing pcDNA3.1-CFP-K_v_7.2 WT or mutants (I340E, W344R) and pcDNA3-HA-K_v_7.3 containing K_v_7.3 with an extracellular hemagglutinin (HA) epitope using FuGENE6 transfection reagent (Promega > 60% transfection efficiency ) for K_v_7.2/K_v_7.3 interaction analysis. Two transfection schemes were used. At first, HEK293 cells were transfected with pcDNA3.1-CFP-K_v_7.2 and pcDNA3-HA-K_v_7.3 at 1:1 ratio (1 μg each), and the expression of CFP-K_v_7.2-W344R was consistently lower compared to WT expression (Supplemental Figure 2B), and its Co-IP with HA-K_v_7.3 was also decreased by 50% compared to CFP-K_v_7.2-WT (Supplemental Figure 2). In order to achieve similar expression levels, we reduced the transfection amounts of pcDNA3.1-CFP-K_v_7.2 WT and I340E plasmids to 0.5 μg for the second sets of Co-IP, while maintaining 1 μg for K_v_7.2-W344R plasmid. At 48 h post transfection, the cells were washed with ice-cold PBS and lysed in ice-cold immunoprecipitation (IP) buffer containing (in mM) 20 Tris-HCl, 100 NaCl, 2 EDTA, 5 EGTA, and 1% Triton X-100 (pH 7.4) supplemented with Halt protease inhibitors (Thermo Fisher Scientific). The cell lysates were incubated on ice for 30 min and supernatant was collected after centrifugation at 14,000×*g* for 15 min at 4 °C. The lysates were first precleared with protein A/G agarose beads (100 μL solution containing 50% beads, Santa Cruz) for 1 h at 4 °C, and then incubated overnight at 4 °C with protein A/G-agarose beads (100 μL) and rabbit anti-HA antibody (1 μL, Cell Signaling, 3724). The small amount of anti-HA antibodies allows us to immunoprecipitate a fraction but not all HA-K_v_7.3 proteins produced in large amount of transfected cells. This way, we can immunoprecipitate the equal amount of HA-K_v_7.3 proteins and examine the effect of mutations on the amount of co-IPed CFP-K_V_7.2 when CFP-K_V_7.2-WT and mutant levels are comparable. After washing with IP buffer to remove nonspecific interactions, the immunoprecipitates were eluted with SDS sample buffer in 1:5 dilution (in mM) containing 75 Tris, 10% SDS, 50 TCEP, 12.5% glycerol, 0.50 EDTA, and 0.50 mg/mL Bromophenol Blue by incubating at 75 °C for 15 min.

### Western blot

Immunoprecipitation samples of the first set of Co-IP (Fig. 1) were fractionated on 6% or 15% SDS-polyacrylamide gels and transferred to polyvinylidene fluoride membranes (PVDF) (Millipore). Membranes were blocked in TBS solution (0.05% Tween-20 in PBS containing 5% milk). Then, they were incubated with the monoclonal primary antibody: anti-K_v_7.2 (1:1000; Neuromab). Secondary antibody was goat anti-mouse IgG horseradish peroxidase conjugate (1:5,000; Bio-Rad). Blots were developed using the Luminata Forte Western HRP substrate reagent (Millipore) and images were digitalized with a Thermo Scientific MYECL Imager. Densitometry of the bands was measured by FIJI software. It was calculated dividing co-immunoprecipitated protein by immunoprecipitated protein, i.e., CFP-K_v_7.2/c-Myc-K_v_7.2.

For the second set of Co-IP (Fig. 2), Western blotting procedure was performed as previously described [3]. Briefly, the lysate and the eluted immunoprecipitates were loaded on 15-well 4–20% SDS-PAGE gels (BioRad) and transferred to a polyvinyl difluoride (PVDF) membrane (Immobilon, Millipore) using wet transfer at 30 mV overnight. Membranes were blocked in blocking buffer (5% milk, 0.1% Tween-20 in TBS) for 1 h and incubated with primary antibodies in washing buffer (1% milk, 0.1% Tween-20 in TBS) overnight at 4 °C. Primary antibodies used are mouse anti-GFP (1:1000 dilution), mouse anti-HA (1:1000 dilution), and rabbit anti-GAPDH antibodies (1:1000 dilution; Cell Signaling, 2955, 2367, 2118). Membranes were then washed with washing buffer and incubated in washing buffer containing donkey anti-rabbit and anti-mouse Horse Radish Peroxidase (HRP)-conjugated secondary antibodies (The Jackson Laboratory, 711–035–152, 715-035-150) at room temperature for 1 h. HRP signals were visualized by Pierce ECL or SuperSignal Pico Plus substrate (Thermo Fisher Scientific #32106, #34577). Western blot images were acquired with the iBright CL1000 imaging system (Thermo Fisher Scientific). Western blot band densities were analyzed using the Image J software (National Institute of Health), normalized to WT CFP-K_v_7.2 with WT being 100%, and presented as % WT. One-way ANOVA with post-ANOVA Tukey multiple comparison tests were conducted using the Origin Pro 2020 software (OriginLab) with a priori value (*p*-value) < 0.05. Data are represented as mean ± standard error of mean (SEM).

### FRET in living cells

Cells were plated at ~ 60% confluence onto 30-mm round coverslips in six-well plates. Cells were transfected as described above. Monomeric CFP and monomeric Citrine were used in these experiments, referred to as CFP and YFP. The N-terminal end of CaM was tagged with mCFP for the indicated experiments. For CFP-CaM binding to YFP-K_v_7.2, YFP-ABCD, or YFP-AB the transfection ratio used was 1:5 with a total of 0.6 μg DNA per M35 dish. For assembly experiments, the ratio was 1:1:2 (0.5 μg of each FCP-tagged ABCD and 1 μg of CaM or 1 μg of empty pcDNA3.1 his/c-Myc vector). Twenty-four hours after transfection, coverslips were placed in an imaging chamber and imaged maintaining them in buffer solution composed of (mM) 140 NaCl, 5 KCl, 1 MgCl_2_, 2 CaCl_2_, 10 glucose, and 10 Na-Hepes, pH 7.4 at room temperature.

Images were recorded using a Nikon D Eclipse TE2000-U fluorescence microscope (Nikon Instruments, Tokyo, Japan) equipped with a confocal scanning head and a spectral detector module. Images were captured using a × 60 oil objective, with the pinhole opened (150 μm) and using the 405 nm laser line (Coherent, Santa Clara, CA, USA) or the 488 nm line (Melles-Griot, Rochester, NY, USA) for direct CFP or YFP excitation, respectively. To assure homogeneity in donor an acceptor expression, cells displaying a signal with emission values between 20 and 170 (arbitrary units) when excited at 405 nm (to record FRET signal) and when excited at 488 nm (to record acceptor emission only) were included in the analysis with FIJI.

The spectral detector allows simultaneous recording of 32 images, each registering a 5-nm band of the spectrum, covering 450–610 nm. After spectral unmixing with EZ-C1 Nikon software, using cells expressing CFP or YFP alone as reference, as described previously [35], the area under the spectra was measured and a FRET index was calculated as FRET index = YFP_405_/CFP_405_, where YFP is the integral of emission signal for YPP_405_, and CFP_405_ is the integral of the emission signal for CFP after excitation with the 405 nm laser line. FRET efficiency was computed from the FRET index using the transfer function:

$$ \mathrm{Efficiency}=0.001+1.0022\ast \left(\mathrm{FRET}\ \mathrm{index}/\left(\mathrm{FRET}\ \mathrm{index}+2.11\right)\right) $$

The parameters were estimated by non-linear fitting (*R*^2^ > 0.99). The relationship between FRET index and FRET efficiency was computed using excitation and emission spectra for donor and acceptor, with a quantum yield of 0.41 for mCFP, and 0.74 for mCitrine and *R*_0_ = 50.26 Å (www.fpbase.org/fret).

### Experimental animals and neuronal culture

All procedures involving animals were reviewed and approved by the Institutional Animal Care and Use Committee at the University of Illinois Urbana-Champaign in accordance with the guidelines of the US National Institutes of Health (protocols 15222). Primary dissociated hippocampal cultures were prepared from 18-day-old embryonic rats and transfected with plasmids (total 0.8 μg) at 5 DIV as described [33].

### Immunocytochemistry

Primary dissociated hippocampal cultures were prepared from 18-day-old embryonic rats, transfected with plasmids (total 0.8 μg) at 5 DIV, and immunostaining for surface and total K_v_7 subunits and axonal initial segment (AIS) markers in hippocampal neurons were performed at 48 h post transfection as described [33]. Fluorescence images were acquired as described [3, 33] using a Zeiss Axio Observed inverted microscope equipped with a Zeiss AxioCam 702 mono Camera and ZEN Blue 2.6 software, and stored with no further modification as CZI and 16-bit TIFF files. Within one experiment, the images were acquired using the same exposure time to compare the fluorescence intensity of the neurons transfected with different constructs.

The background-subtracted mean fluorescence intensity of the soma, the axon within 0–30 μm of the beginning of the axon (AIS), the axon between 50 and 80 μm from the beginning of the axon (distal axon), and the major primary dendrites were quantified using ImageJ Software (National Institutes of Health) as described [33].

### Translation analysis

The DNAs cloned in pProHex-HTc corresponding WT and W344R CRD flanked by blue mTFP1 and yellow mcpVenus fluorophores in the N- and C-termini respectively were transformed in *E. coli* BL21 cells alone or together with the pOKD4 plasmid carrying the CaM gene. Cells were grown over night at 37 °C and diluted into 20 ml of fresh LB for further growing at 37 °C till OD_600_ 0.6. Protein expression was induced during 3 h at 37 °C or overnight at 18 °C by addition of 1 mM IPTG. Cells were harvested by centrifugation at 7000 rpm for 10 min. The cell pellets were resuspended in lysis buffer 50 mM Hepes, pH 7.4, 120 mM KCl, 5 mM NaCl, 5 mM EGTA, 1 mM DTT, and protease inhibitors (1X Complete; Roche Applied Science), and similar OD values were fitted for all the samples. The cellular cultures were sonicated 3 times, 5 s on, 5 s off, and centrifuged at 19,000×*g* during 30 min for supernatant and pellet separation. The pellets were resuspended in the same buffer volume used before. Protein solubility was studied by SDS-PAGE electrophoresis (10%) using unboiled samples, by analyzing the protein amount present in the same volume of pellet and supernatant fractions. The gels were visualized using Versadoc imaging equipment, exciting using blue or green LEDs, combined with 530BP28 or 605BP35 emission filters. The protein amount in the pellet and in the supernatant was estimated relative to the total protein amount by quantification of the gel bands using the ImageJ software. The protein soluble fractions were also analyzed in a Fluoromax-3 fluorimeter by recording the emission spectra of mTFP1 and mcpVenus fluorescent proteins upon excitation at 458 and 515 nm, respectively. FRET index was established as the ratio of emission at 520–525 divided by emission at 485–490 nm upon excitation at 456–460 nm. FRET efficiency was computed from the FRET index using the transfer function:

$$ \mathrm{Efficiency}=-0.9279+1.9335\ast \left(\mathrm{FRET}\ \mathrm{index}/\left(\mathrm{FRET}\ \mathrm{index}+0.6821\right)\right) $$

The parameters were estimated by non-linear fitting (*R*^2^ > 0.99). The relationship between FRET index and FRET efficiency was computed using excitation and emission spectra for donor and acceptor, with a quantum yield of 0.85 for mTFP1, and 0.64 for mcpVenus and R_0_ = 59.82 Å (www.fpbase.org/fret).

### Urea mediated protein denaturalization and renaturalization

K_v_7.2 WT and W344R cloned in pProHex-HTc were transformed in *E. coli* BL21 cells in the absence of CaM and grown over night at 37 °C. Cell cultures were diluted into 20 ml of fresh LB for further growing at 37 °C till OD_600_ 0.6. Protein expression was induced during 3 h at 37 °C by addition of 1 mM IPTG. Cells were harvested by centrifugation at 7000 rpm for 10 min and resuspended in 500 μl lysis buffer 120 mM KCl, 50 mM Hepes, pH 7.4, 1 mM PMSF, 1% Triton, and protease inhibitors. The cellular cultures were sonicated 3 times, 5 s on, 5 s off, and centrifuged at 19,000×*g* during 30 min for supernatant and pellet separation. The pellets were resuspended in 120 mM KCl, 50 mM Hepes, pH 7.4, and protease inhibitors (buffer A) and centrifuged at 19,000×*g* for 30 min. The pellets were resuspended in buffer A supplemented with 1 M urea and incubated for 20 min. Samples were centrifuged again at 19,000×*g* for 30 min and resuspended in buffer A containing 6 M urea for protein extraction. After 30 min incubation at 4 °C, the solubilized proteins were collected by centrifugation at 19,000×*g* during 30 min and diluted to 0.1 mg/ml. Diluted proteins were dialyzed in the absence and presence of CaM against buffer 120 mM KCl, 50 mM Hepes, pH 7.4, 5 mM DTT, and urea decreasing concentrations. Proteins were finally dialyzed in 120 mM KCl, 50 mM Hepes, pH 7.4, 5 mM NaCl, 5 mM EGTA, and 5 mM DTT for further fluorescence recordings.

### Force profile analysis

The sequence coding for the SecM arresting peptide (AP) FSTPVWISQHAPIRGSP was inserted between helix B and mcpVenus into the DNAs cloned in pProHex-HTc corresponding to WT and W344R CRD flanked by mTFP1 and mcpVenus fluorophores in the N- and C-termini respectively. The sequence (EFYVGYVPGGSPGRPGGSRPHVGSGGQQGSHV) of the linker joining helix B with SecM AP contained restrictions sites that allowed creating a library with 22, 27, 34, 40, and 50 amino acids. Note that the AP residues are computed. Two additional constructs with linker lengths of 18 and 24 residues were generated by introducing novel restrictions sites at the desired positions. The constructs were transformed in *E. coli* BL21 cells alone or together with the pOKD4 plasmid carrying the CaM gene. Single colonies were used to start overnight cultures, which were induced and processed as described in translation analysis. The protein soluble fractions were also analyzed in a Fluoromax-3 fluorimeter by recording the emission spectra of mTFP1 and mcpVenus fluorescent proteins upon excitation at 458 and 515 nm, respectively.

### Stability calculations

Binding affinities have been computed for five different mutations at position 344 using the *Rosetta Flex ddG* [48] prediction protocol for the CaM/K_v_7.2-hAB complex (PDB: 6FEG [13]). In short, WT and mutant models are generated by performing random displacements of the protein backbone named “backrub moves” in a shell of 8 Å around the mutation site. Side chains of both WT and mutants are optimized by assigning a score to each mutation, based on the all-atom Rosetta Energy Function 2015 [49]. The resulting score associated to the mutation is compared with the WT to compute *ΔΔG = ΔG*_*Mutation*_ *− ΔG*_*WT*_. Therefore, if *ΔΔG* > 0 the WT would show a stronger binding affinity compared to the mutated one, and the opposite for *ΔΔG* < 0. Following Rosetta’s protocol, fifty different simulations were performed for each mutant, and each one consists of 50,000 backrub moves. These random moves are accepted or rejected based on the Metropolis criterion with an energy of 1.2 kT, and the final value of the binding affinity is the mean value of all 50 simulations. The backrub moves constitute a random trajectory in the angles and provides a sampling of possible configurations of the residue 344 side chain. For each move the free energy difference (*ΔG*) is calculated considering the Rosetta energy function 2015 and its final value is the mean value of all 50 simulations.

Additionally, as atomic coordinates are saved every 5000 steps and each simulation generates 10 snapshots of the trajectory, we used the VMD interface together with the CHARMM36 all-atom force field [62] to analyze in detail the structural characteristics of the simulations.

Finally, molecular dynamics simulations were carried out of the WT and mutant helices A, TW, and B with the software NAMD 2.13 and the CHARMM36 all-atom force field. The input structure was PDB 6FEG [13] eliminating the N-terminal residual amino acids up to residue number 328. The simulation was performed in a periodic cubic box of TIP3P water so that the minimum distance of any protein atom and the edge of the box was at least 6.1 Å. A concentration of 120 mM of KCl and 5 mM of NaCl was introduced to mimic neuron physiology. SHAKE bond length constraints were applied to all bonds, nonbonded interactions were calculated by the particle-mesh Ewald method with a cutoff of 12 Å. The simulation was first minimized using 1000 steepest descent steps; after that, 0.5 ns were simulated in the canonical ensemble at 298 K, keeping the same temperature using Langevin dynamics with a Langevin damping of 0.5 ps^−1^. After that, 1.5 ns of NPT ensemble was simulated in order to accommodate the periodic cell and avoid the formation of vacuum bubbles in the solvent with a pressure target of 1 atmosphere. Finally, 100 ns were simulated in NPT ensemble for both WT and W344R mutant systems.

### Statistical analysis

Values are presented as the mean ± SEM. The differences between the means were evaluated using the unpaired Student’s *t*-test or ANOVA with Mann-Whitney post hoc on SigmaStat Statistic (SigmaPlot 11), where values of *p* < 0.05 were considered significant. The number of cells in each experiment is indicated in brackets in the figures. The results are from two or more independent batches of cells. In all figures an asterisk, double asterisks, and triple asterisks indicate significance at *p* < 0.05, *p* < 0.01, and *p* < 0.001, respectively.

Images from hippocampal neurons were analyzed using Origin 9.1 (Origin Lab), the Student’s *t*-test and one-way ANOVA with post-ANOVA Tukey’s and Fisher’s multiple comparison tests were performed to identify the statistically significant difference with a priori value *p* < 0.05 between two groups and for greater than three groups, respectively.

## Supplementary Information

**Additional file 1: Figure S1.** Relationship between normalized current densities from cells expressing Kv7.2 channels carrying the indicated mutations in the helix A. **Figure S2.** The W344R mutation reduced expression of CFP-Kv7.2 in cultured hippocampal neurons. **Figure S3.** I340E and W344R mutants severely reduced surface and total expression of heteromeric HA-Kv7.3/CFP-Kv7.2 in the axons of cultured hippocampal neurons. **Figure S4.** Background-subtracted fluorescent intensities of surface HA-Kv7.3 in different compartments of cultured hippocampal neurons. **Figure S5.** Cartoon representation of a Kv7 channel. **Figure S6.** Emission spectra of the purified mTFP1-AB-mcpVenus/CaM complex in the presence of increasing concentrations of the denaturant urea. **Figure S7.** Emission spectra of the soluble WT and W344R mTFP1-AB-mcpVenus proteins translated in CaM-free non-denaturing conditions. **Figure S8.** Fluorescent image of a SDS-PAGE of unboiled bacterial extracts of cells expressing WT or W344R mTFP1-AB-mcpVenus proteins, expressed at 18°C. **Figure S9.** Schematic representation of the constructs used for in vivo translation and representative fluorescent images of SDS-PAGE gels loaded with unboiled bacterial extracts expressing WT-AP construct with and without CaM. **Figure S10.** Relationship between current densities of homomeric Kv7.2 channels carrying the indicated mutations at position 344 and the computed binding energies in Rosetta Energy Units. **Figure S11.** Time series of the angle of Tryptophan 344 through a molecular dynamics simulation of the Kv7.2 WT CRD forced to start in T configuration.

**Additional file 2.** Data on which the conclusions rely.

## Data Availability

All data generated or analyzed during this study are included in this published article and its supplementary information files.
