# Supplementary material for: An epilepsy-causing mutation leads to co-translational misfolding of the Kv7.2 channel
Source: BMC Biol. 2021 May 21;19:109. doi: 10.1186/s12915-021-01040-1 (PMC8138981; doi:10.1186/s12915-021-01040-1)
Supplement: Supplementary file 1 — Additional file 1: Figure S1. Relationship between normalized current densities from cells expressing Kv7.2 channels carrying the indicated mutations in the helix A. Figure S2. The W344R mutation reduced expression of CFP-Kv7.2 in cultured hippocampal neurons. Figure S3. I340E and W344R mutants severely reduced surface and total expression of heteromeric HA-Kv7.3/CFP-Kv7.2 in the axons of cultured hippocampal neurons. Figure S4. Background-subtracted fluorescent intensities of surface HA-Kv7.3 in different compartments of cultured hippocampal neurons. Figure S5. Cartoon representation of a Kv7 channel. Figure S6. Emission spectra of the purified mTFP1-AB-mcpVenus/CaM complex in the presence of increasing concentrations of the denaturant urea. Figure S7. Emission spectra of the soluble WT and W344R mTFP1-AB-mcpVenus proteins translated in CaM-free non-denaturing conditions. Figure S8. Fluorescent image of a SDS-PAGE of unboiled bacterial extracts of cells expressing WT or W344R mTFP1-AB-mcpVenus proteins, expressed at 18°C. Figure S9. Schematic representation of the constructs used for in vivo translation and representative fluorescent images of SDS-PAGE gels loaded with unboiled bacterial extracts expressing WT-AP construct with and without CaM. Figure S10. Relationship between current densities of homomeric Kv7.2 channels carrying the indicated mutations at position 344 and the computed binding energies in Rosetta Energy Units. Figure S11. Time series of the angle of Tryptophan 344 through a molecular dynamics simulation of the Kv7.2 WT CRD forced to start in T configuration. [file 12915_2021_1040_MOESM1_ESM.pdf]

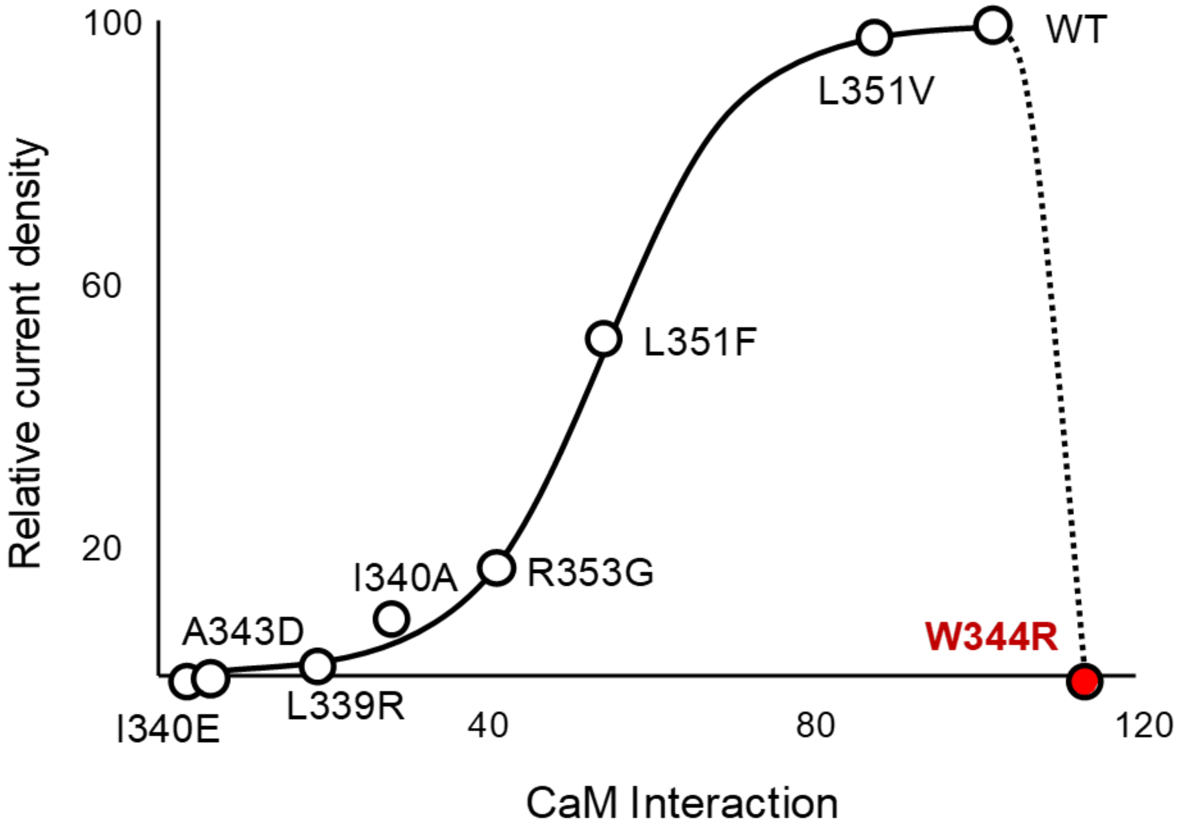

Supplemental Figure 1. Relationship between normalized current densities from cells expressing $K_V7.2$ channels carrying the indicated mutations in the helix A segment co-expressed with $K_V7.3$ subunits (y axis) and maximal fluorescent emission (x axis) of dansylated CaM when bound to recombinant AB proteins carrying the indicated proteins. The W344R mutant deviates from the relationship. Adapted from [30; 31].

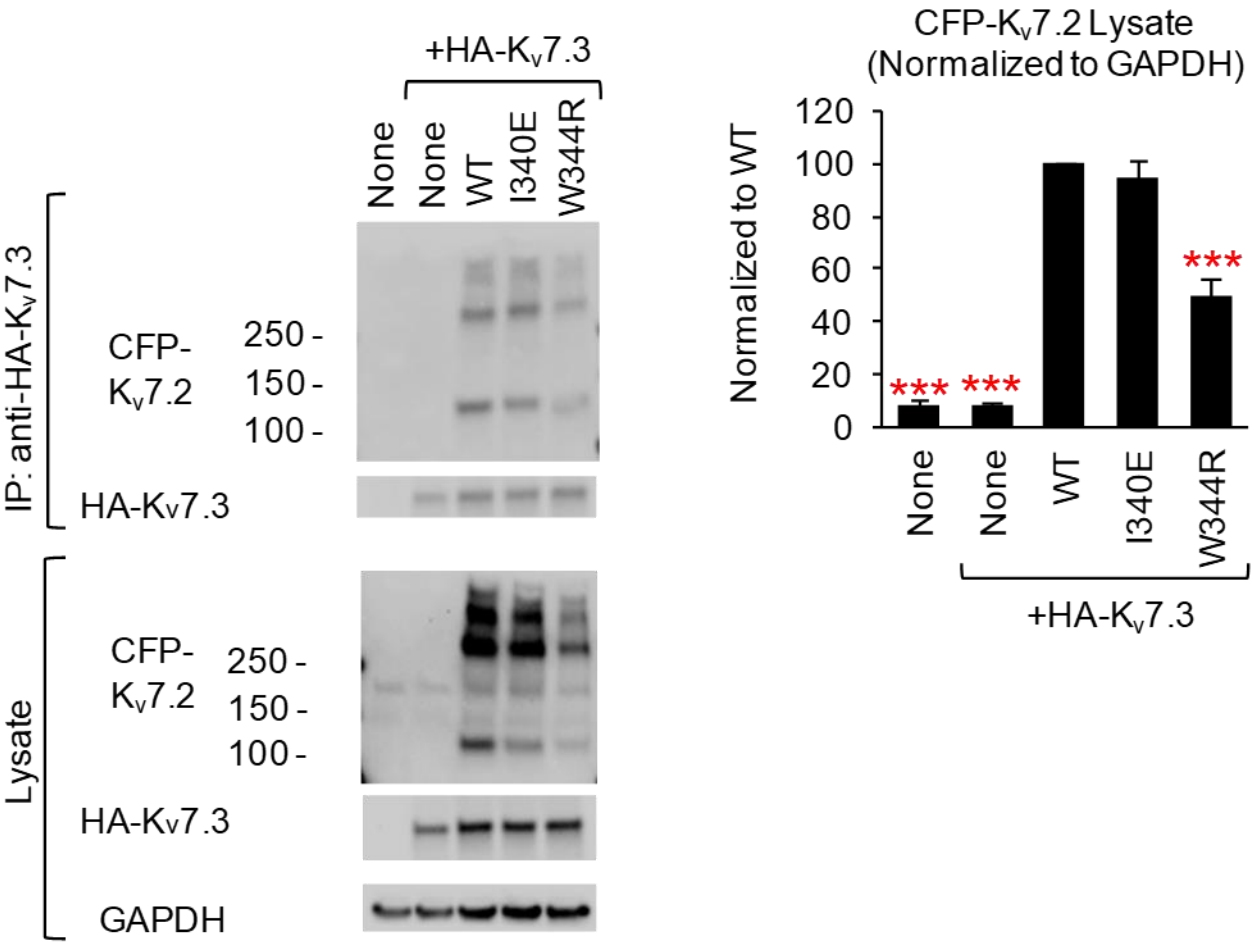

Supplemental Figure 2, related to Figure 2. The W344R mutation reduced expression of CFP-Kv7.2. A Representative immunoblots of CFP-Kv7.2 lysates in HEK293T cells co-transfected with 1 µg of pcDNA3-HA-Kv7.3 and 1 µg pcDNA3.1-CFP-Kv7.2 WT or I340E or W344R. All immunoblots of CFP-Kv7.2 showed monomers (~130 kD), dimers (~260 kD), trimers (~390 kD), and tetramers (~520 kD) even though the immunoprecipitated fractions and lysates were treated with SDS sample buffer and strong reducing agent TCEP. The trimeric and tetrameric bands were not separated well due to their large size. **B.** Quantification of immunoblots (n = 5). GAPDH served as a loading control and all samples were normalized to WT with WT being 100%. Data represents the mean ± SEM (*p < 0.05, ***p < 0.005 vs CFP-Kv7.2 WT).

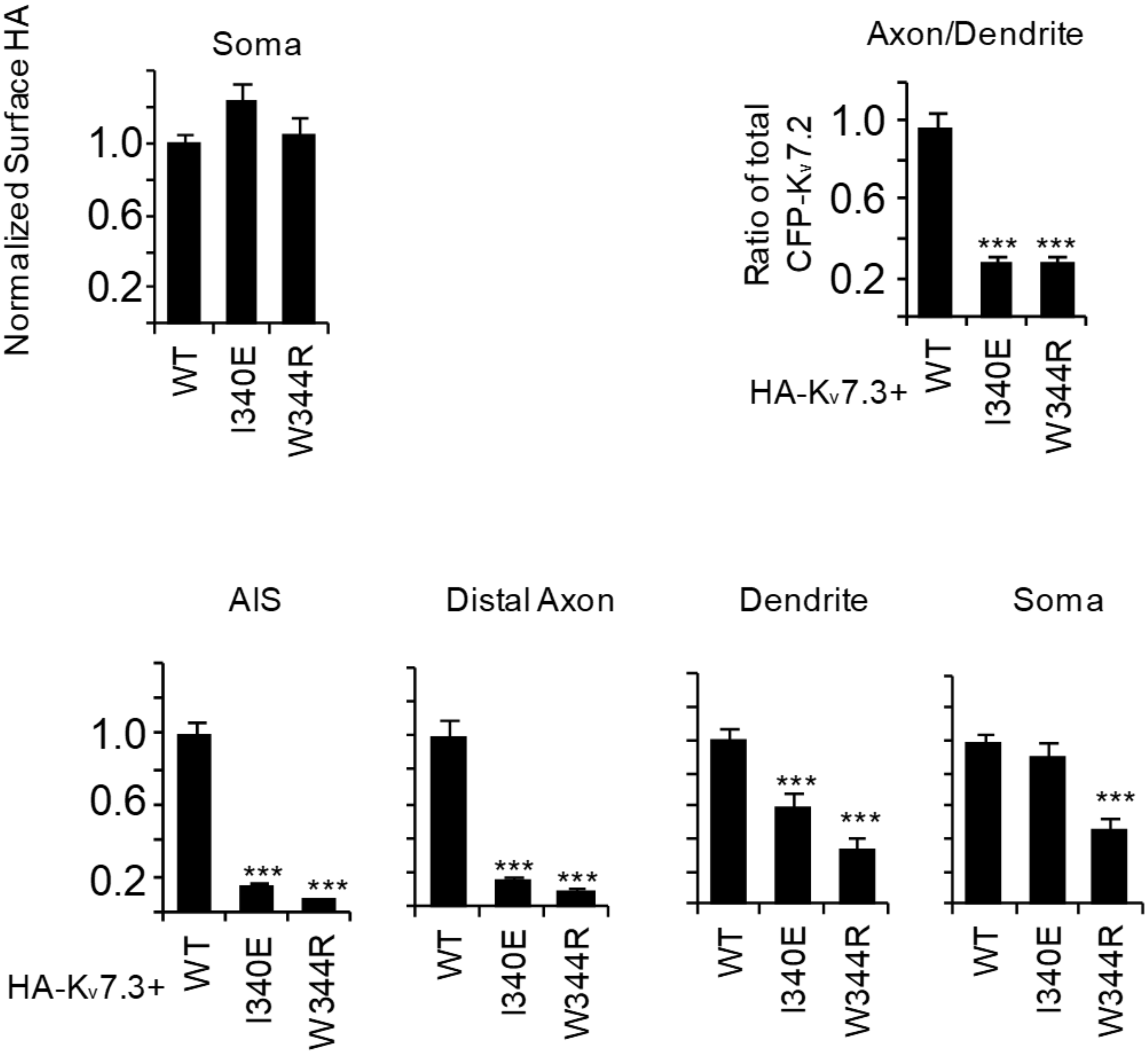

Supplemental Figure 3, related to Figure 3. I340E and W344R mutants severely reduced surface and total expression of heteromeric HA-K_v7.3/CFP-K_v7.2 in the axons of cultured hippocampal neurons. A. Background-subtracted fluorescent intensities of surface HA-K_v7.3 from transfected neuronal soma were normalized to those of HA-K_v7.3/CFP-K_v7.2-WT. **B-C** Total expression of CFP-K_v7.2 from transfected neurons. **B**. The Axon/Dendrite ratio was computed for total CFP-K_v7.2. **C**. Background-subtracted fluorescent intensities of total CFP-K_v7.2 were normalized to those of WT. Sample numbers are: WT (n = 19), I340E (n = 14), and W344R (n = 13). Data represent Ave ± SEM. One-way ANOVA with post-hoc Fisher's LSD test was conducted. ***$p < 0.005$.

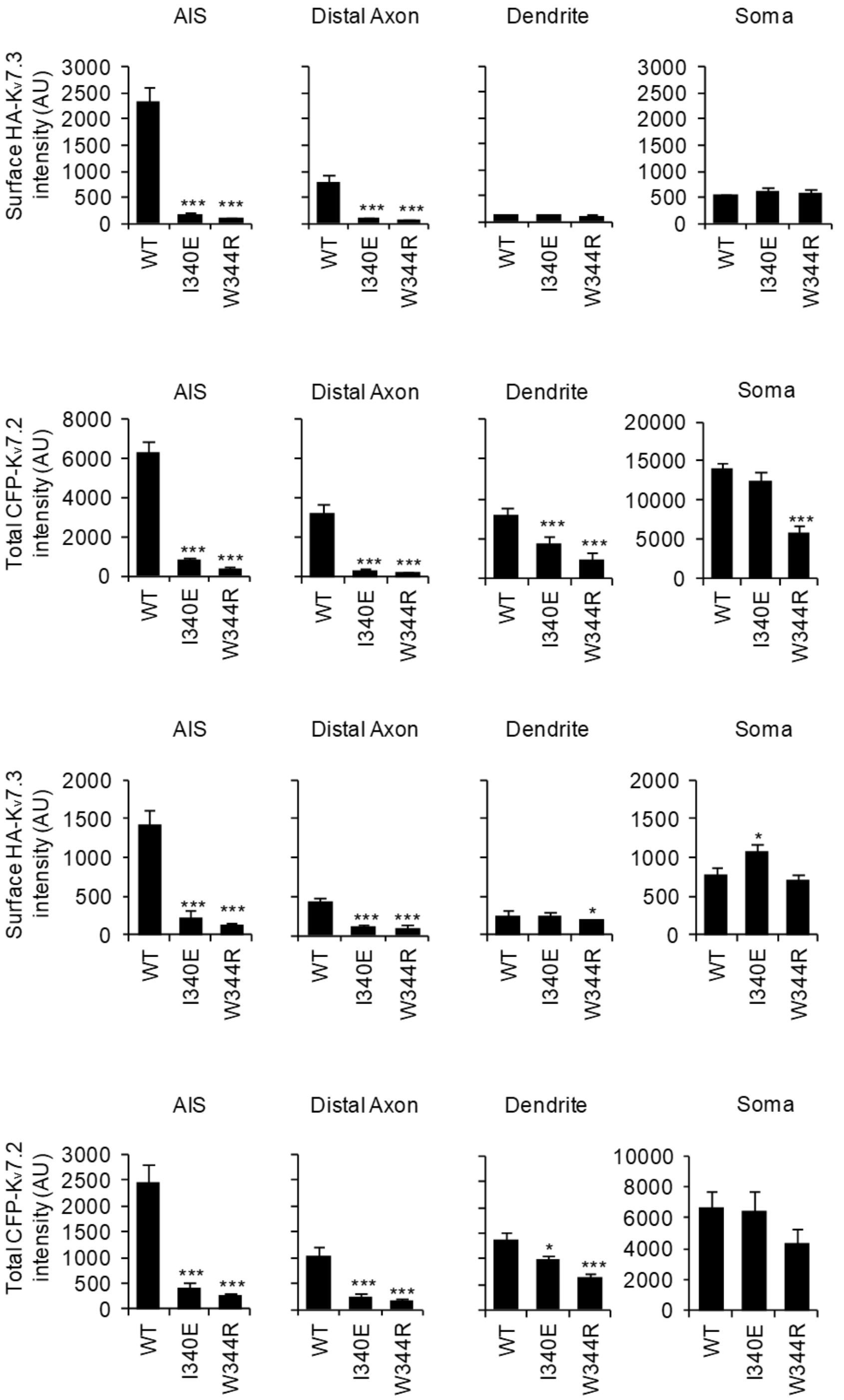

Supplemental Figure 4, related to Figure 3. Background-subtracted fluorescent intensities of surface HA-K_V7.3 (**A, C**) and total CFP-K_V7.2 (**B, D**) from two individual experiments: experiment 1 (A-B), experiment 2 (C-D). Sample numbers are: (A-B) WT (n = 14), I340E (n = 9), and W344R (n = 10); (C-D) WT (n = 5), I340E (n = 5), and W344R (n = 3). Data represent Ave ± SEM. One-way ANOVA with post-hoc Fisher's LSD test was conducted. *$p < 0.05$ ***$p < 0.005$.

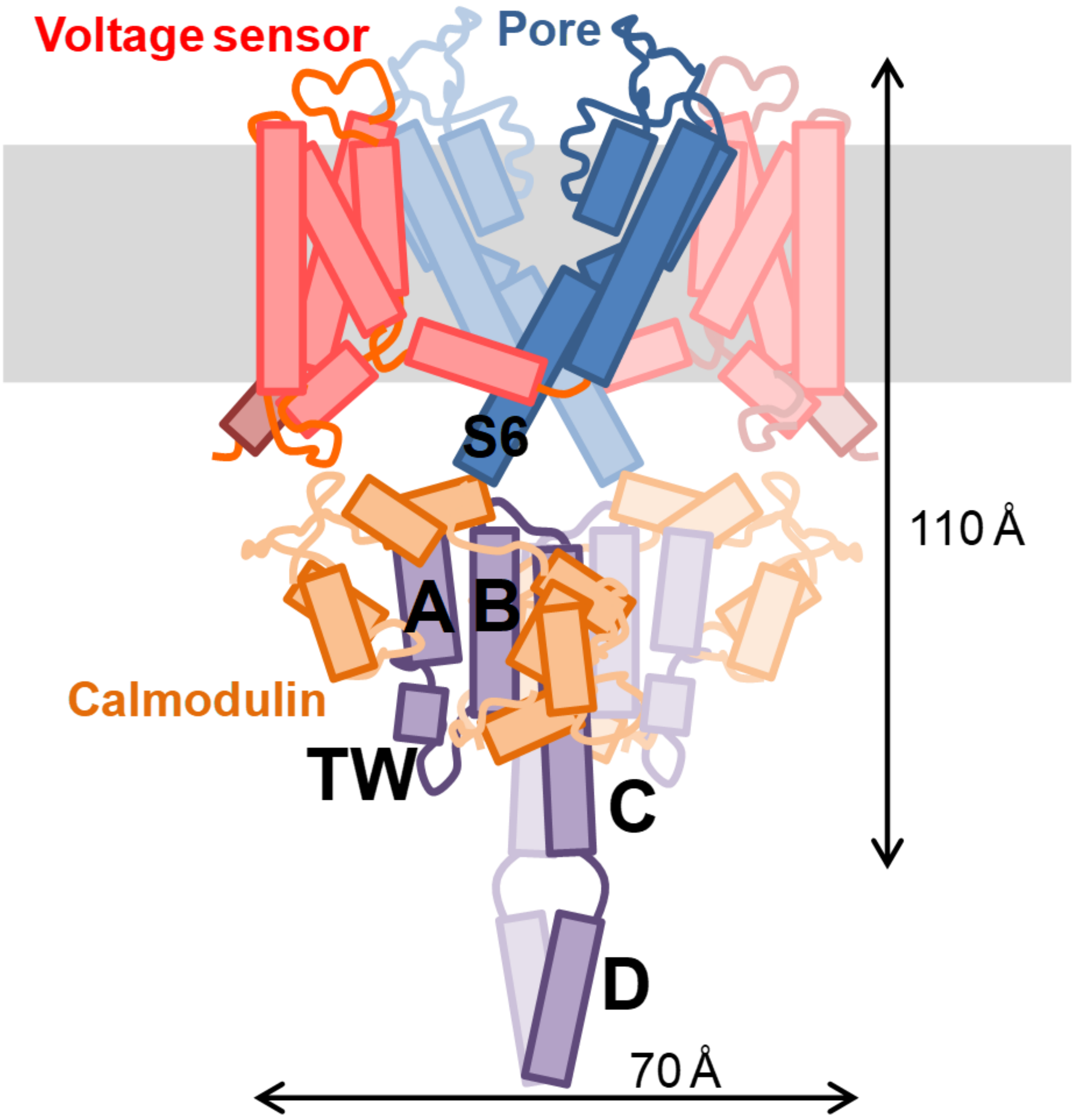

Supplemental Figure 5, related to Figure 4. Cartoon representation of a K_v7 channel. Only two subunits of the tetrameric assembly are drawn for clarity. The ABCD domain resembles a flower bouquet with the coiled-coil helix D corresponding to the pedestal.

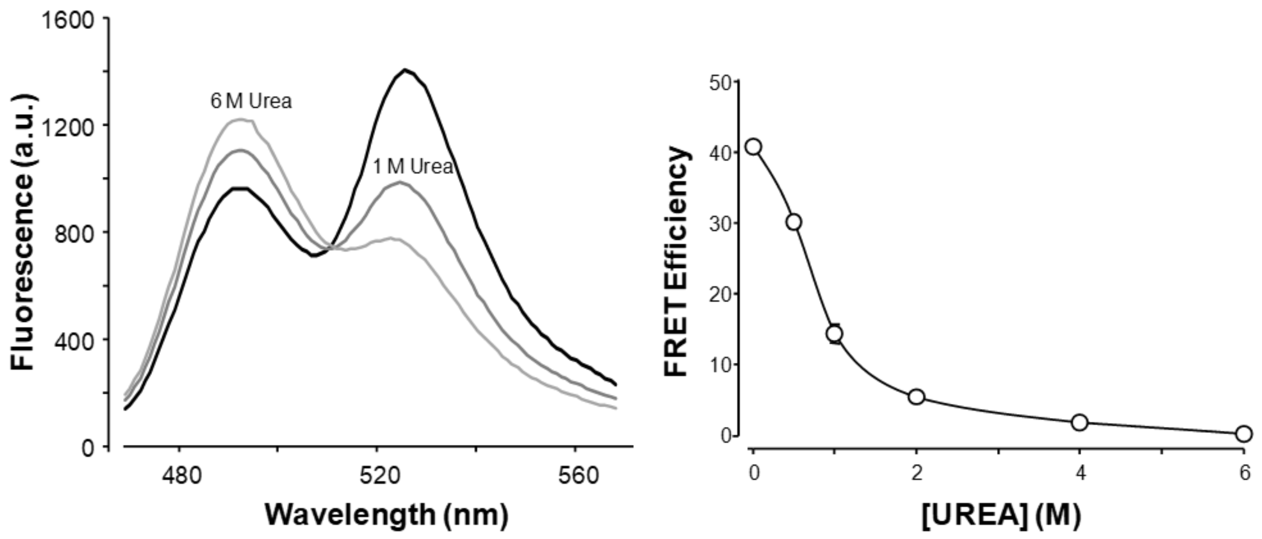

Supplemental Figure 6, related to Figure 5. A. Emission spectra of the purified mTFP1-AB-mcpVenus/CaM complex in the presence of increasing concentrations of the denaturant urea. **B.** FRET efficiency values computed from the ratio mcpVenus/mTFP1 peak emission (528/492 nm) ratio from spectra as a function of urea concentration (n = 4).

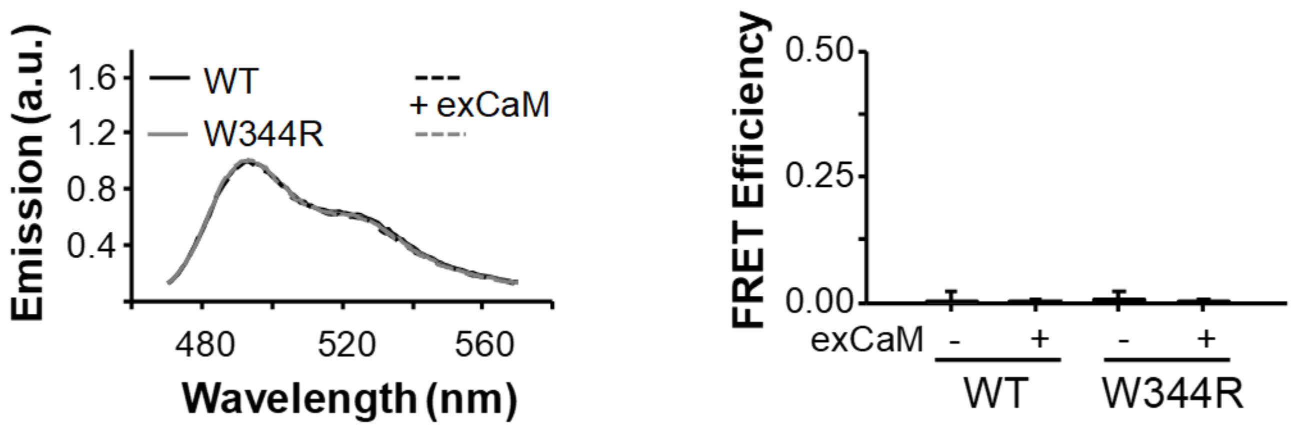

Supplemental Figure 7, related to Figure 6. A. Emission spectra of the soluble WT (black lines) and W344R (grey lines) proteins at ~6 µM translated in CaM-free non-denaturing conditions. An excess (100 µM) exogenous-CaM (exCaM) was added to each sample (dotted lines) and the emission spectra were measured after 24 hours (n = 4). **B.** FRET efficiency values computed from the ratio mcpVenus/mTFP1 peak emission (528/492 nm) from spectra as in A.

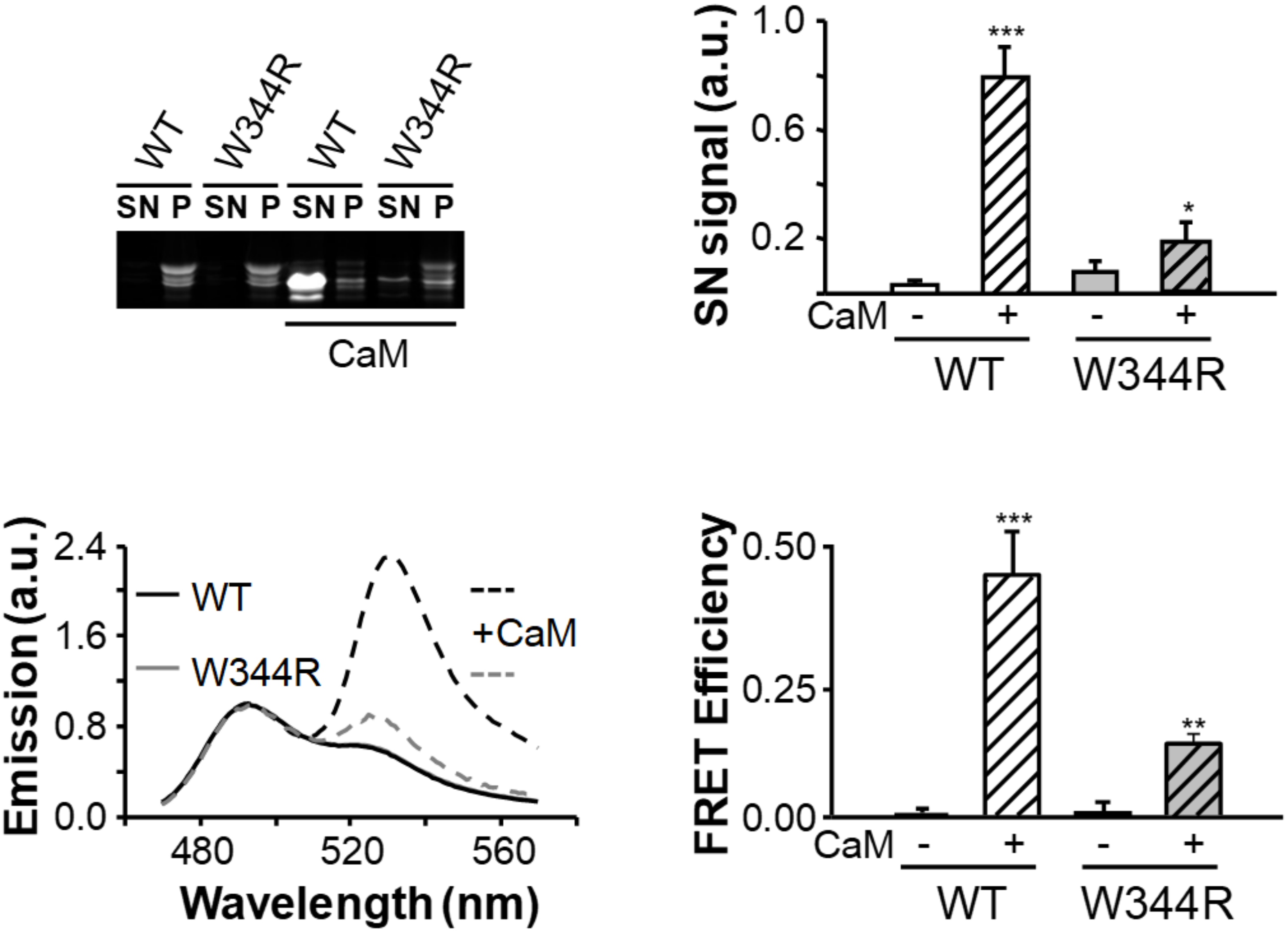

Supplemental Figure 8, related to Figure 6. A. Fluorescent image of a SDS-PAGE of unboiled bacterial extracts of cells expressing WT or W344R biosensors, expressed at 18°C. Proteins were co-expressed (right columns) or not (left columns) with CaM. Soluble (supernatant; SN) and insoluble (pellet; P) protein fractions were separated, and loaded as indicated. **B.** Fluorescence intensity of the supernatant band of SDS-PAGE gel (n = 4). **C.** Emission spectra of the soluble fraction of WT (black lines) and W344R (grey lines) proteins expressed alone (solid lines) or co-expressed with CaM (dashed lines). **D.** FRET efficiency values computed from the ratio YFP/CFP peak emission (528/492 nm) ratio from spectra as in C (n = 4).

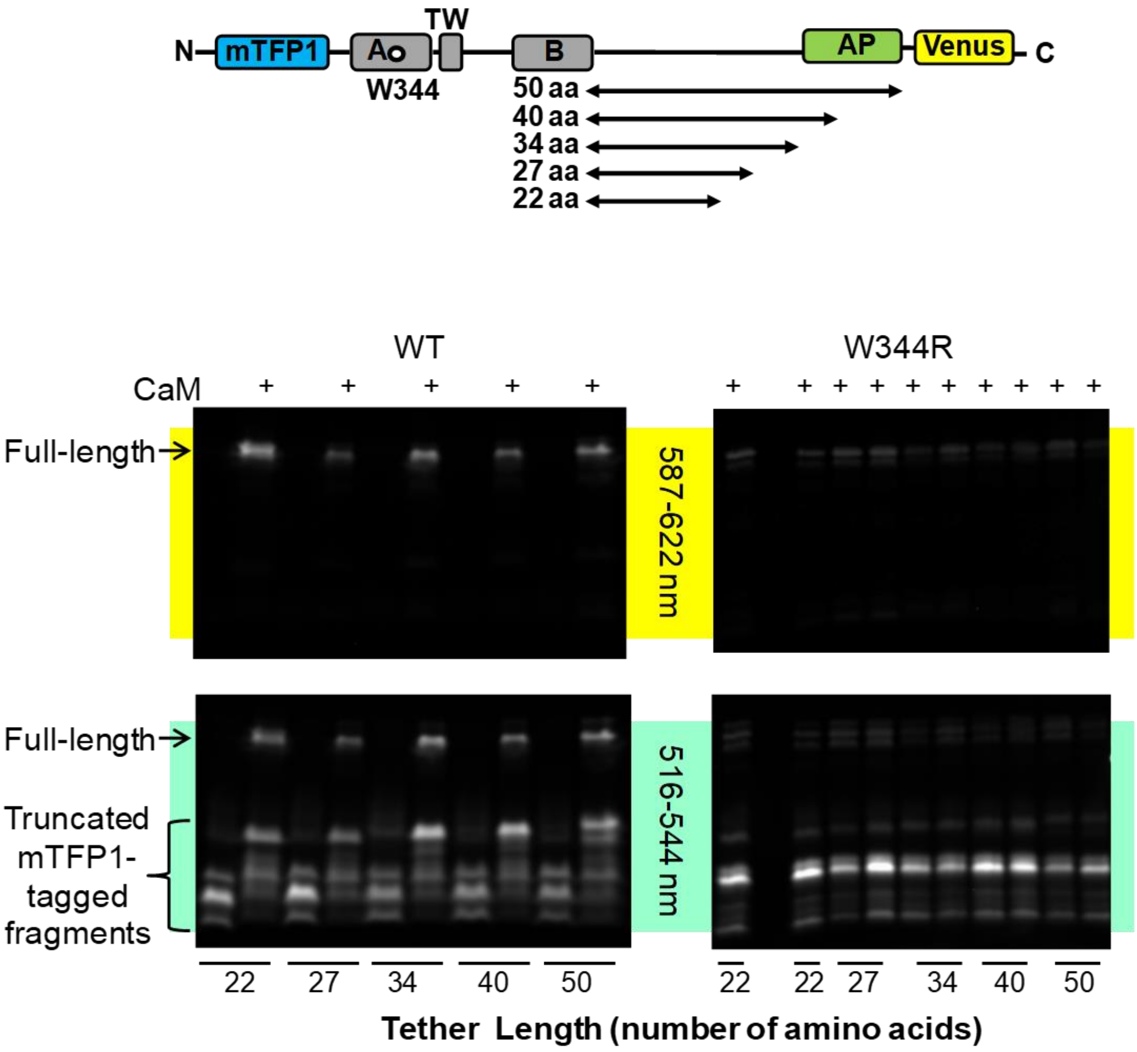

Supplemental Figure 9, related to Figure 6. A. Schematic representation of the constructs used for *in vivo* translation. The CRD was cloned upstream of the SecM arresting peptide (AP) sequence with tethers of increasing length, ranging from 22 to 50 amino acids from the C-terminal conserved Pro of the SecM AP where translational stalling takes place. **B.** Representative fluorescent images of SDS-PAGE gels loaded with unboiled bacterial extracts expressing WT-AP construct with and without CaM, with tether lengths indicated at the bottom. A 605BP35 filter was used to isolate emission from mcpVenus on the image at the top, whereas the image at the bottom, a 530BP28 filter was used to detect emission from both mTFP1 and mcpVenus. **C.** Fluorescent images as in B of bacterial extracts expressing W344R-AP constructs in the presence of CaM. The second line was not loaded.

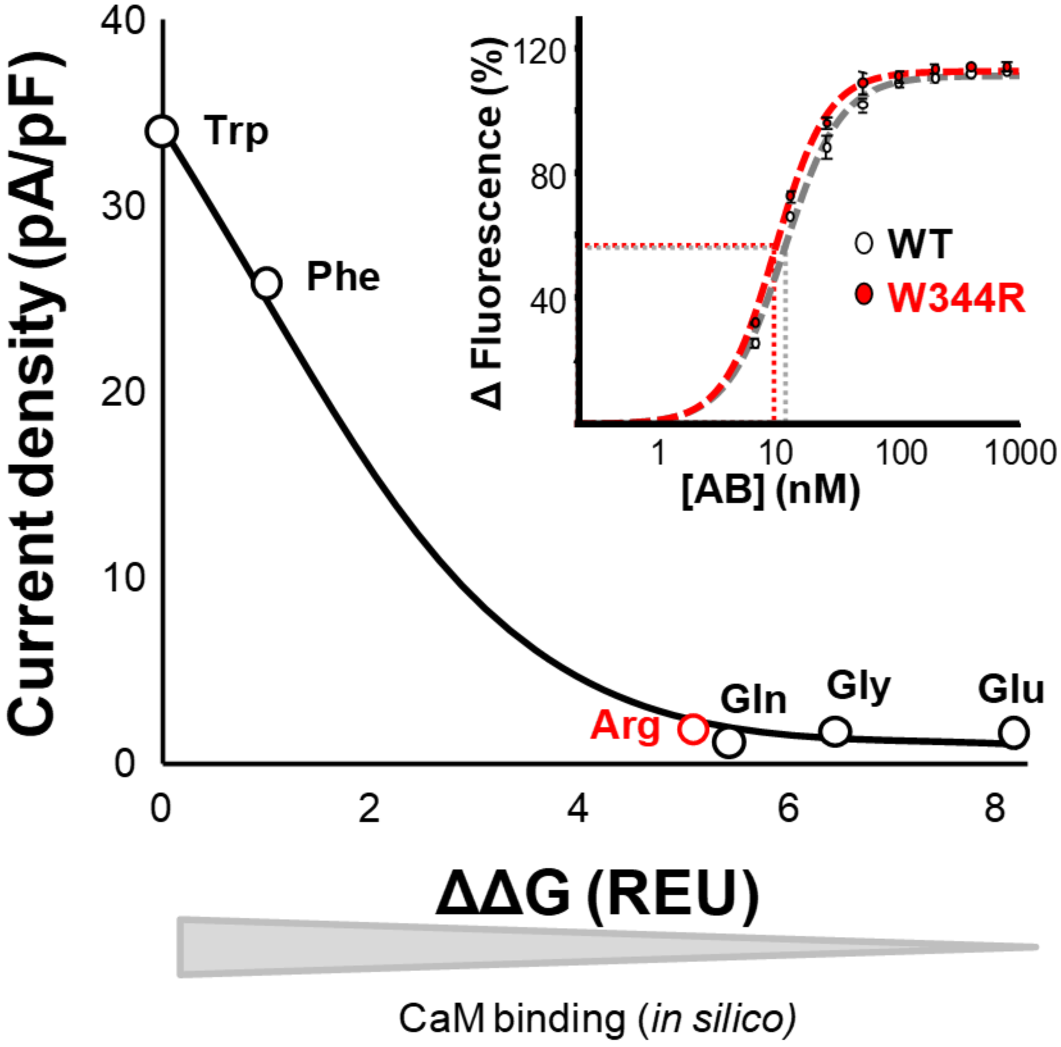

Supplemental Figure 10, related to Figure 7. Relationship between current densities of homomeric $K_V7.2$ channels carrying the indicated mutations at position 344 and the computed binding energies in Rosetta Energy Units (REU). It is expected that the higher the value, the weaker the predicted affinity for CaM. However, the measured apparent binding affinity is 13% more favorable for the W344R than WT (Inset, from [30]).

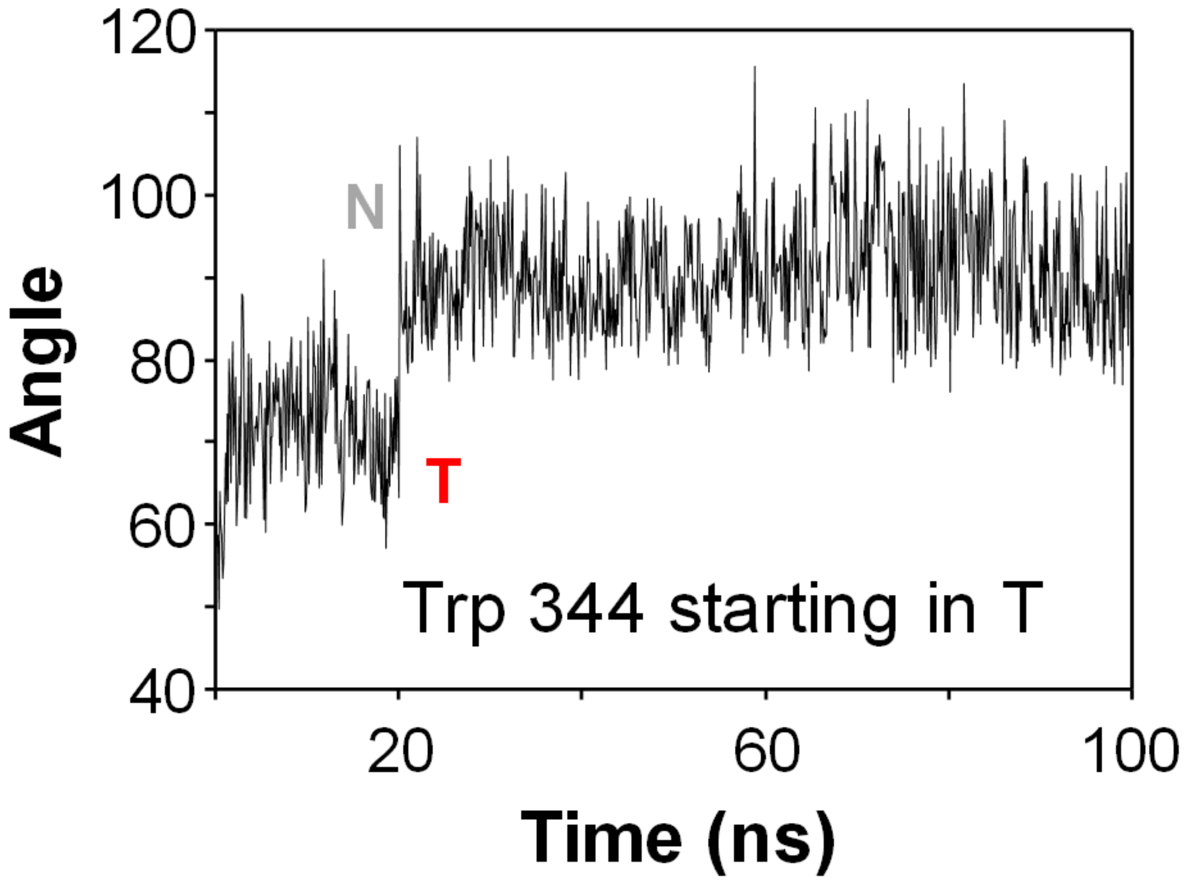

Supplemental Figure 11, related to Figure 7. Time series of the angle of Tryptophan 344 through a molecular dynamics simulation of the $K_v7.2$ WT CRD forced to start in T configuration. Note that for the first 20 ns, tryptophan 344 remains in T and then exhibits a conformational change towards the more stable N configuration.
